# Governing Generative Artificial Intelligence in Anatomy Education: A Proposed Risk-Tiered Framework for Donor-Derived Material, Anatomical Fidelity, and Assessment

**DOI:** 10.7759/cureus.115656

**Published:** 2026-09-02

**Authors:** Ahmed Mahgoub, Anaida Singh, Ayman Mahgoub

**Affiliations:** 1 Anatomy, University of Khartoum, Khartoum, SDN; 2 Anatomical Sciences, St. George's University, St. George's, GRD; 3 Research and Development, Anova Health, Riyadh, SAU

**Keywords:** anatomy education, artificial intelligence governance, assessment, body donation, educational technology, generative artificial intelligence, human oversight, risk classification

## Abstract

Generative artificial intelligence (GenAI) is now routinely used in anatomy education to draft explanations, generate and interpret images, produce assessment items, and support feedback. Broad responsible-AI principles are well established, but they do not resolve the questions anatomy educators actually face: whether donor-derived material may be entered into an external system, what counts as adequate verification of a generated anatomical structure, and which assessment decisions must remain human. Anatomy faculty themselves report unclear governance in these areas and ask for discipline-specific policy. This article proposes an operational governance framework for GenAI in anatomy education, developed from a structured synthesis of 71 sources spanning direct anatomy studies, transferable health professions education (HPE) evidence, professional guidance, and ethical scholarship. Five discipline-specific pressure points motivate the proposal: donor-derived inputs retain stewardship obligations after digitization; anatomical fidelity is relational and structural rather than stylistic, so fluency and realism are not evidence of correctness; representation and clinically relevant variation form part of anatomical truth; assessment converts content error into consequential judgment about learners; and provenance, intellectual property, and disclosure support traceability and accountability. The framework governs the defined use case rather than the AI product. Purpose, audience, input and data class, consequence, scale, and degree of AI autonomy determine one of four proposed tiers (Low, Moderate, High, and Restricted/Avoid) under a conservative rule in which the highest triggered criterion sets the tier. Permitted uses then pass five control gates covering inputs and provenance, anatomical fidelity, variation and representation, assessment validity where applicable, and disclosure and accountability. Four decisions remain non-delegable to a machine, and approval is treated as a revisable state maintained through a monitoring, incident-response, and revalidation loop rather than a one-time authorization. The framework is an evidence-informed proposal for local adaptation, not a validated instrument; it has not been prospectively evaluated, its tier thresholds are not empirically calibrated, and local law, donor-program policy, and institutional governance remain controlling.

## Introduction and background

Generative artificial intelligence (GenAI) is increasingly being studied and used in anatomy education. Large language and multimodal models can generate explanations and assessment items, create or interpret visual content, support feedback, and assist educational and research tasks [1]. Recent anatomy-education literature has examined both the potential utility of these systems and concerns regarding factual accuracy, reliability, transparency, bias, over-reliance, and the preservation of human judgment [1-3].

In this article, GenAI refers to artificial intelligence systems capable of generating new content, including text or images, in response to user inputs. This distinction matters because not all evidence relevant to GenAI governance comes from generative systems. This review focuses on GenAI; evidence from non-generative AI is included selectively when it provides directly transferable evidence for governance questions such as automated assessment, human oversight, or accountability, and such evidence is identified as transferable where it is used.

The need for discipline-specific governance is not merely theoretical. In a cross-sectional survey of 30 anatomy faculty at medical and health sciences colleges in the United Arab Emirates, 28 participants (93.3%) reported using GenAI. The most frequently reported educational uses were generating quiz or examination questions, summarizing complex topics, and drafting instructional materials; image or diagram generation was less common [4]. Faculty-identified needs included clearer governance and disclosure policies and safeguards for assessment [4]. The small sample limits generalizability, but the findings provide direct evidence that governance uncertainty is being reported by anatomy educators themselves rather than inferred on their behalf.

Anatomy presents a combination of governance questions that take distinctive forms in the discipline. Five pressure points recur. Donor-derived material raises questions about whether particular images or data may legitimately enter an external GenAI system. Generated explanations and images require verification of anatomical accuracy in their structural and relational detail rather than evaluation based on fluency or visual plausibility alone. Learner-facing resources raise related but distinct questions about clinically relevant anatomical variation and demographic or phenotypic representation. GenAI involvement in assessment can introduce errors into processes that carry consequences for learners. Finally, provenance, permissions, material AI contribution, and accountable human ownership must remain sufficiently traceable to support audit and accountability. These five concerns - donor-derived material, anatomical fidelity, variation and representation, assessment, and provenance and disclosure - form the discipline-specific pressure points developed in this article.

Existing responsible-AI frameworks provide substantial foundations for addressing these questions. Health CARE-AI offers consensus-based guidance for responsible AI use across health professions education (HPE), research, and care, emphasizing professional and institutional accountability, human judgment, appropriate information use, equity, and transparency [5]. The National Institute of Standards and Technology (NIST) Generative Artificial Intelligence Profile provides cross-sector guidance for identifying and managing GenAI risks across the AI lifecycle [6]. FUTURE-AI provides lifecycle-oriented recommendations for trustworthy AI in healthcare, spanning design, validation, deployment, and monitoring [7]. The United Nations Educational, Scientific and Cultural Organization (UNESCO) provides human-centered guidance specifically for GenAI in education and research, including privacy, human agency, validation, and institutional policy [8], while the Organisation for Economic Co-operation and Development (OECD) AI Principles address human-centered values, transparency and explainability, robustness and safety, traceability, and accountability [9].

These instruments establish substantial cross-cutting principles, risk-management processes, lifecycle expectations, and accountability structures. They do not, however, resolve several discipline-specific determinations required in anatomy education: which donor-derived inputs may legitimately enter a GenAI system, what constitutes adequate verification of generated anatomy, how variation and representation should be evaluated, which assessment uses require heightened control, and which decisions should remain human-accountable. An anatomy-specific operational layer is therefore needed to translate cross-cutting guidance into discipline-specific decisions.

Relevant operational approaches also exist within HPE. Masters' AMEE Guide No. 158 addresses the ethical use of AI in HPE [10], while Gin et al. adapt the established concept of entrustment to AI and propose graduated levels of AI participation and human supervision for specific educational tasks [11]. These are important antecedents to the present proposal and mean that task-specific governance, graduated oversight, and retained human responsibility should not be presented as conceptually unique to the proposed framework.

The contribution proposed here is narrower and discipline-specific. Among the 71 sources retained for framework development, we did not identify a single framework integrating all five anatomy-specific pressure points into an operational approach linking use-case classification, authorization, verification, human accountability, and continuing monitoring. This observation is bounded by the review's search strategy and retained evidence set and should not be interpreted as evidence that no other relevant framework exists.

This article therefore proposes an anatomy-specific operational layer that builds on, rather than replaces, existing responsible-AI and HPE guidance. The defined educational use case - not the AI product in the abstract - is characterized by its purpose, audience, input and data class, consequence, scale, and degree of AI autonomy. These characteristics inform a proposed risk tier, after which relevant control gates, retained human decisions, and lifecycle monitoring are applied. The framework addresses GenAI uses initiated or authorized by educators and governed at course, departmental, or institutional level. Learner-originated uses outside those governed workflows, including independent upload of donor-derived material to consumer GenAI systems, fall outside the operational scope of the proposed framework, although institutions remain responsible for establishing policies governing such conduct. Learner-originated GenAI use is therefore identified as a priority for further governance research. The proposed framework is intended for local adaptation by anatomy programs and institutions. It has not been prospectively evaluated, and its tier thresholds have not been empirically calibrated. It is not a substitute for applicable law, donor-program policy, institutional governance, or regulatory compliance.

## Review

Evidence identification and framework development

Design and Rationale

This article uses a structured, evidence-informed state-of-the-art review with framework development. It was not designed as a systematic or scoping review, and no claim of compliance with the Preferred Reporting Items for Systematic Reviews and Meta-Analyses (PRISMA) is made. The evidence base spans empirical anatomy studies, reviews, transferable HPE research, professional and policy guidance, and conceptual or ethical scholarship. These sources serve different evidentiary functions and cannot appropriately be pooled or appraised using a single methodological instrument without obscuring those differences [12,13].

The purpose of the review was therefore not to estimate a pooled effect or provide an exhaustive map of all potentially relevant literature. Rather, it was to identify evidence relevant to recurrent governance problems in anatomy education, characterize the directness and limitations of that evidence, and use the resulting synthesis to develop an operational framework while distinguishing the evidence from the author-developed governance components informed by it.

Evidence Sources, Search Scope, and Dates 

Evidence identification was organized through six prespecified streams. PubMed/MEDLINE provided the structured bibliographic component. Google Scholar was used as a supplementary discovery and verification source. Professional, institutional, publisher, and DOI sources were consulted where relevant. The original documentation preserves the purpose, search date, concept blocks, and discovery or verification sources for each stream. The historical record does not preserve a separate formal backward- or forward-citation-chasing procedure or source-specific yield; none is reconstructed here. The six prespecified evidence streams, their search dates, purposes, concept blocks, and discovery or verification sources are summarized in Table 1.

**Table 1:** Search-stream documentation The table provides a structured, evidence-informed state-of-the-art review with framework development. The final substantive evidence set comprised 71 retained sources. GenAI-focused literature was emphasized from 2022 through August 8, 2026; older foundational anatomy and donor-ethics sources were retained where relevant. Exact historical query strings and complete per-stream retrieval, deduplication, screening, and exclusion counts were not prospectively archived and have not been retrospectively reconstructed. Accordingly, this table documents the historical search process at stream level and should not be interpreted as a PRISMA-style record flow. No separate formal citation-chasing stream or yield is reconstructed from the historical record. The organizational sources named in the table are those preserved in the historical stream documentation. WHO and ICMJE documents are retained in the 71-source evidence matrix, but the surviving record does not preserve a source-specific retrieval route for those documents; no such route is reconstructed here. AI: artificial intelligence; MCQ: multiple-choice question; OECD: Organisation for Economic Co-operation and Development; OSPE: objective structured practical examination; NIST: National Institute of Standards and Technology; UNESCO: United Nations Educational, Scientific and Cultural Organization; PRISMA: Preferred Reporting Items for Systematic Reviews and Meta-Analyses; WHO: World Health Organization; ICMJE: International Committee of Medical Journal Editors; IFAA: International Federation of Associations of Anatomists; AAMC: Association of American Medical Colleges

Stream	Search date	Prespecified purpose	Concept blocks used	Discovery and verification sources
S1 - Core anatomy and GenAI	Aug 7, 2026	Identify anatomy-specific GenAI and AI sources directly addressing or materially informing governance, ethics, oversight, reliability, and implementation.	Anatomy education; generative AI/large language models; ethics; governance; reliability; oversight; implementation	PubMed/MEDLINE; Google Scholar; publisher and DOI verification; professional and institutional sources where relevant
S2 - Donor-derived materials and digital governance	Aug 7, 2026	Identify anatomy and human-remains evidence on donor consent, provenance, digital images, derivatives, privacy, ownership, and external dissemination.	Body donation; cadaveric/human-tissue images; consent; dignity; provenance; digitization; privacy; ownership; dissemination	PubMed/MEDLINE; Google Scholar; IFAA; Human Tissue Authority; publisher and DOI verification
S3 - Anatomical accuracy and verification	Aug 7, 2026	Identify direct anatomy evidence on factual accuracy, hallucination, multimodal recognition, citation reliability, model comparison, and verification.	Anatomy; factual accuracy; hallucination; image recognition; multimodal AI; citations/references; model comparison; verification	PubMed/MEDLINE; Google Scholar; publisher and DOI verification
S4 - Visual fidelity and variation/representation	Aug 7, 2026	Determine whether visual fidelity and representation require separate but linked controls and identify evidence concerning anatomical variation, representation, and bias.	AI-generated images; medical/anatomical illustration; anatomical fidelity; variation; representation; demographic bias	PubMed/MEDLINE; Google Scholar; publisher and DOI verification
S5 - Assessment	Aug 7, 2026	Define controls for formative versus summative use, AI-generated items, automated scoring, practical/OSPE applications, psychometrics, feedback, and human oversight.	Anatomy assessment; MCQ/item generation; OSPE/practical assessment; automated scoring; psychometrics; feedback; human oversight	PubMed/MEDLINE; Google Scholar; publisher and DOI verification
S6 - Governance and accountability	Aug 8, 2026	Define accountability, authorization, documentation/disclosure, monitoring, re-evaluation, incident response, withdrawal, and revalidation.	Responsible AI; governance; accountability; authorization; monitoring; incident response; revalidation; disclosure; institutional policy	PubMed/MEDLINE; Google Scholar; NIST; UNESCO; AAMC; OECD; publisher and DOI verification

GenAI-focused literature was emphasized from 2022 through 8 August 2026. Older sources were retained where required to address foundational donor ethics, human-tissue imagery, anatomy education, or professional responsibilities that predate contemporary GenAI.

The primary structured bibliographic search was limited principally to PubMed/MEDLINE. A prospectively defined broader database set including Scopus, Web of Science, Embase, ERIC, IEEE Xplore, or ACM Digital Library was not searched. This limitation is acknowledged explicitly below.

Exact historical search strings were not prospectively archived, and complete retrieval, deduplication, screening, and exclusion counts cannot be reconstructed reliably. Those values are therefore not estimated or inserted retrospectively. The evidence-identification process is auditable at the level of the six documented streams and retained source matrix, but it is not independently reproducible as a systematic search.

Regulatory instruments discussed later in the article were verified against authoritative legal sources during manuscript revision and are treated as contextual legal references rather than being retrospectively inserted into the historical 71-source framework-development set.

Eligibility and Evidence Inclusion

Sources were retained when they made a substantive contribution to an anatomy-relevant governance question. Eligible evidence therefore included direct anatomy empirical research and reviews; transferable HPE evidence where anatomy-specific evidence was limited; professional, consensus, policy, regulatory, or standards-based guidance; and conceptual or ethical scholarship relevant to donor stewardship, provenance, accountability, or related governance boundaries.

The review focuses on generative AI. Non-generative AI evidence was retained selectively where it addressed a directly transferable governance problem, particularly automated assessment, human oversight, supervision, or accountability. Such evidence is identified as transferable rather than presented as evidence of GenAI performance.

Older literature concerning body donation, cadaveric imagery, consent, dignity, provenance, privacy, and stewardship remained relevant because these responsibilities predate contemporary GenAI.

The final eight governance domains were not used as a retrospective eligibility criterion. Sources were first retained because they substantively informed the review question and were subsequently coded to a principal governance domain or as cross-cutting. This distinction avoids defining eligibility by the framework that the retained evidence was itself being used to develop.

To clarify the scope of the final evidence set without retrospectively reconstructing a PRISMA-style screening history, the revised synthesis treats as outside scope: reports concerned only with clinical-care or research AI when no transferable educational-governance implication was identified; conference abstracts; and preprints subsequently superseded by a peer-reviewed report, for which the published version is used. Non-generative AI was not excluded categorically when it provided directly transferable governance evidence. These statements describe the scope of the final synthesis and are not presented as prospectively archived exclusion rules or as a basis for reconstructing exclusion counts.

Methodological, definitional, background, and regulatory-context citations used to explain the review design or contemporary legal environment are also distinguished from the 71 substantive framework-development sources.

Selection, Coding, and Evidence Classification

The final retained evidence base comprised 71 substantive sources: 25 direct anatomy sources, 24 transferable HPE sources, 13 professional or normative sources, and 9 conceptual or ethical works. Each retained source was assigned an evidence-basis category and a principal governance domain or was coded as cross-cutting. The complete source-level coding is provided in the Appendices.

Screening and source coding were performed by one author (AM). No independent double-screening or duplicate source coding was undertaken, and no formal inter-rater reliability assessment was performed. The categorizations should therefore be interpreted as a transparent single-rater synthesis rather than independently replicated classifications.

The working evidence matrix contains fields for relevance, source credibility, and framework utility. Their exact historical operational role cannot now be reconstructed with sufficient confidence. They are therefore not used in the revised article as inclusion or exclusion criteria, evidence weights, certainty ratings, or determinants of recommendation strength.

Appraisal and Evidentiary Strength

No single formal risk-of-bias instrument was applied across the retained set, and no GRADE-style certainty assessment was undertaken. The heterogeneous source types were instead appraised descriptively according to their design, setting, task, sample or output set where applicable, and relevance to the governance question.

Among the 25 direct anatomy sources, 20 were original empirical studies, two systematic reviews, one scoping review, one critical review, and one other educational source. The empirical literature included bounded model-output evaluations, expert-rating studies, authentic learner cohorts, psychometric studies, content analyses, automated assessment applications, and repeated comparisons across model versions.

Study scale varied substantially, ranging from bounded model-output evaluations to authentic learner cohorts of several hundred students. Preregistration status was not systematically captured or verified in the evidence matrix and therefore was not used as an appraisal criterion.

The evidence is unevenly distributed across governance domains. Direct anatomy evidence is concentrated most heavily in anatomical fidelity and assessment; the representation domain relies on transferable HPE evidence, while human oversight and institutional lifecycle governance are also supported predominantly by transferable and professional sources.

These evidence profiles are descriptive rather than quantitative certainty judgments. Proposed controls should not be interpreted as having been prospectively validated simply because the failure mode they address is documented empirically.

Five discipline-specific pressure points

Donor-Derived Inputs Retain Stewardship Obligations After Digitization

Photographs, videos, scans, and derived representations of donated bodies remain connected to the consent arrangements, institutional responsibilities, and stewardship expectations under which the material was obtained. Professional guidance represented within the retained evidence emphasizes appropriate consent or permission, protection against identification, controlled distribution, secure storage, provenance, dignity, and institutional oversight [14-18].

The literature does not establish one universal rule governing every use of donor-derived imagery. The framework therefore does not create a universal consent rule. Before donor-derived material is processed through GenAI, the responsible program should establish its provenance, applicable consent or permission scope, intended purpose, relevant law and institutional policy, and the nature of the proposed platform exposure.

De-identification also requires more than removal of names or electronic metadata. Potentially identifying features of donor-derived images may include facial structure, tattoos, scars, surgical hardware, dentition, unusual pathology, visible labels, or other distinctive physical characteristics. The proposed review of these features is an author-developed operational safeguard, not a validated donor-image de-identification instrument.

Where removal of potentially identifying characteristics would materially compromise educational value, de-identification should not simply be assumed to resolve the governance problem; the use should instead be escalated to the appropriate donor-program or institutional authority.

A conservative default is also proposed for external or commercial platforms: donor-derived imagery should not be uploaded to or processed by an external commercial GenAI system unless institutional authorization has been obtained and relevant contractual and technical safeguards have been reviewed. Zero-retention or no-training commitments may reduce particular risks but do not independently establish permission for donor-image processing.

Relevant platform review may include data retention, use for model training or service improvement, secondary processing, subcontractor or third-party access, storage arrangements, deletion provisions, security controls, and contractual responsibility. Such review should involve the relevant anatomy or donor-program authority together with appropriate institutional privacy, information-security, legal, compliance, or equivalent expertise rather than an individual educator acting alone.

Independent learner use of consumer GenAI outside educator- or institution-authorized workflows is outside the operational tiering scope of this framework. Institutions should nevertheless address learner photography, upload, transformation, or circulation of donor-derived material through applicable laboratory, donor, professionalism, privacy, and AI-use policies. Learner-originated use remains an important research and policy priority.

Anatomical Fidelity Is Structural and Relational, Not Stylistic

Anatomical correctness depends on structure identity, position, course, attachment, branching, laterality, orientation, and spatial relationships. A fluent explanation or visually persuasive image may therefore remain anatomically incorrect.

Direct anatomy studies demonstrate task-, model-, modality-, version-, and workflow-dependent limitations in generated anatomical content, image interpretation, and question answering [19-25]. The governance implication is that the specific output requires verification rather than reliance on model reputation, aggregate benchmark performance, fluency, or photorealism.

Source reliability is a separate fidelity problem. Anatomy-specific evaluation has documented hallucinated, inaccurate, or unsupported references [23]. A bibliographically plausible citation supplied by a GenAI system should therefore not be accepted without locating the underlying publication and confirming that it supports the associated claim.

Recognition should also be distinguished from subsequent reasoning. Where a task depends on identification of an anatomical image or structure before interpretation or explanation, an upstream recognition error should not be obscured by plausible downstream reasoning [24].

Model performance can also change over time. Comparisons across successive model versions show that aggregate improvement does not establish equal reliability across topics or cognitive levels [25]. Validation of one provider, model version, modality, prompt structure, retrieval system, or workflow should therefore not be generalized automatically to a materially different configuration.

The proposed control is qualified anatomical verification before authoritative learner-facing use. Relevant structural claims should be checked against appropriate independent authoritative sources. Visual content should be examined for identity, laterality, orientation, spatial relationships, omissions, additions, labels, and clinically meaningful departures from accepted anatomy. Final acceptance of authoritative anatomy remains human-accountable.

Anatomical Variation and Demographic Representation Are Related but Distinct

Two related but different issues should be distinguished: clinically relevant anatomical variation and demographic or phenotypic representation.

Clinically relevant anatomical variation concerns meaningful departures from the most commonly taught arrangement that may affect anatomical understanding or clinical reasoning, including vascular, biliary, urinary, neural, and other variations. Direct evidence evaluating whether GenAI-generated educational anatomy reliably represents such variants remains very limited. The framework should therefore not claim that GenAI systematically omits clinically relevant variants. Instead, the absence of direct evidence itself constitutes an important research gap.

Demographic and phenotypic representation concerns whether generated visual material unduly narrows the range of bodies represented or reproduces stereotyped patterns. The retained evidence supporting this concern is principally transferable from medical-education and clinical image-generation studies. Alon et al.'s study, for example, is a systematic literature review rather than an individual image-generation experiment [26].

Learner-facing visual resources may therefore require two different forms of review. Individual images should satisfy anatomical-fidelity requirements, while sets of images may require review for unjustifiably narrow demographic or phenotypic representation. Where clinically relevant anatomical variation is part of the educational objective, important variants should be considered explicitly rather than treating a single configuration as universal.

The representation component of this proposed gate is informed predominantly by transferable empirical evidence. The anatomical-variation component is an evidence-informed precautionary safeguard in an area where direct anatomy-specific GenAI evidence remains sparse.

Assessment Converts Content Error Into Consequential Judgment

GenAI can assist assessment-item development, but item generation does not itself establish validity. Appropriate evaluation may include anatomical and factual accuracy, alignment with learning objectives and blueprint, intended cognitive level, item-writing quality, fairness, and post-use psychometric performance.

Kıyak et al. evaluated expert-reviewed, department-approved AI-generated anatomy questions administered to 502 second-year medical students, reporting usable item difficulty and discrimination characteristics [27].

Lopiano et al. evaluated ChatGPT-4 in gross anatomy and embryology. Initial prompting generated 300 questions, with 80.0% judged accurate and 7.7% higher order; modified prompting generated a further 210 questions, with 70.5% judged accurate and 41.0% higher order [28]. This represents a trade-off observed in one study, rather than a general law that increasing higher-order generation necessarily reduces accuracy.

Tekin et al. generated 240 anatomy questions using GPT-4, selected 30 for administration to 280 students, and reported acceptable internal consistency, while expert agreement with GPT-4's cognitive-level classification was only 26.6% for the administered questions [29]. Such findings illustrate why satisfactory psychometrics cannot substitute for content and cognitive-level review.

Performance on image-dependent practical tasks is also variable. Meo et al. evaluated Google Bard on morphological, histopathological, and radiological image-identification tasks using an OSPE-type format, providing transferable evidence that image-dependent assessment performance should not be inferred from text-only performance [30].

Automated assessment evidence also requires modality-specific interpretation. Bernard et al. evaluated decision-tree methods rather than GenAI for grading a 54-question anatomy OSPE using data from 368 learners and reported high local grading accuracy [31]. This is relevant anatomy evidence concerning automated assessment governance but should not be represented as direct GenAI evidence.

The proposed framework therefore allows GenAI to assist with question drafting, formative material, feedback, and defined components of assessment workflows where appropriate human review and task-specific validation are present. Summative or consequential uses warrant stronger content and blueprint review, quality assurance (QA), psychometric or performance monitoring where appropriate, and a named accountable human decision-maker.

The proposed boundary that final grading, progression, pass/fail, disciplinary, or contested high-stakes judgments remain human-accountable is an evidence-informed precautionary governance position, not an empirically calibrated GenAI threshold.

Provenance, Intellectual Property, and Disclosure Support Traceability and Accountability

A fifth pressure point concerns whether the origin, transformation history, permission status, material AI contribution, and accountable ownership of educational material remain sufficiently traceable.

Provenance concerns the origin and history of material. Permission and intellectual-property considerations concern whether content may appropriately or lawfully be reused, transformed, or redistributed. Disclosure concerns whether material GenAI involvement is communicated proportionately and whether accountable human ownership remains identifiable.

Ho et al. examined 239 English-language YouTube videos relevant to digital anatomy education and identified a substantial gap between ethical expectations and observed reporting of provenance and consent [32]. That finding should not be generalized to all anatomy resources, but it demonstrates that loss of traceability in digital educational practice is an observed problem.

Cornwall et al. discuss legal and ethical questions associated with using existing anatomical illustrations to create new illustrations, including permission, attribution, and derivative use [33]. Similar questions arise when GenAI systems transform or build upon existing educational resources.

The framework therefore proposes four linked requirements: document source and provenance when material to the use case; verify applicable permissions and attribution; disclose meaningful AI involvement proportionately; and identify a responsible human approver.

These recommendations should not be converted into universal copyright conclusions. Copyright, licensing, derivative-use rights, ownership, and contractual requirements vary by jurisdiction, source material, platform terms, and publication context.

Disclosure should likewise be proportionate. Reusable learner-facing content, formal instructional exemplars, publications, or consequential assessment workflows may warrant substantially more explicit disclosure than private brainstorming or routine wording assistance.

Framework development and derivation of the governance domains

The final framework contains eight governance domains, but the five anatomy-specific pressure points do not correspond to eight independent anatomy-specific failure modes.

Five domains arise directly from the discipline-specific pressure points: donor-derived material maps to Domain 2 (input and donor-derived material governance); anatomical fidelity to Domain 3 (anatomical fidelity and source verification); anatomical variation and representation to Domain 4 (variation, bias, and representation); assessment to Domain 5 (assessment validity and integrity); and provenance and disclosure to Domain 6 (provenance, intellectual property, and disclosure).

Three additional domains provide the operational governance architecture. Domain 1 - use-case and risk classification - is a cross-cutting author-developed classification layer. Only one retained source was coded principally to this domain, although several retained governance sources provide cross-cutting support. Domain 7 - human oversight and accountability - and Domain 8 - institutional authorization, monitoring, and incident response - provide cross-cutting governance mechanisms supported predominantly by transferable HPE and professional or normative evidence.

The resulting architecture is therefore best understood as five anatomy-specific problem domains plus one cross-cutting classification layer plus two cross-cutting governance layers. The evidence profile, synthesis rationale, minimum proposed control, status, and principal limitation for each of the eight governance domains are summarized in Table 2.

**Table 2:** Evidence landscape across eight governance domains “Principal” refers to the governance domain to which a retained source was primarily coded; eight additional sources were coded as cross-cutting. Status codes describe evidentiary provenance rather than certainty: E = empirical evidence documents the relevant failure mode or rationale; G = professional or normative guidance supports the principle; P = the operational rule, threshold, or boundary is author-proposed. Combined codes indicate mixed provenance. E does not mean that the proposed control itself has been prospectively validated. *For Domain 4, the empirical evidence is predominantly transferable rather than anatomy-specific. HPE: health professions education

Governance domain	Evidence profile	Synthesis rationale	Minimum proposed control	Status	Principal limitation
1. Use-case and risk classification	One principal direct-anatomy source; additional cross-cutting governance support	Governance should be tied to the defined use case rather than to the AI product alone.	Define purpose, audience, input/data class, consequence, scale, and permitted autonomy before authorization.	P	Classification construct is author-developed and unvalidated.
2. Input and donor-derived material governance	Eight sources coded principally to this domain: four professional/normative, three conceptual/ethical, one direct anatomy	Digitization does not extinguish consent, dignity, provenance, privacy, or stewardship considerations.	Establish provenance and permission; review identifying features and platform authorization before processing.	G/P	Law, consent, and institutional requirements differ; operational safeguards are not validated instruments.
3. Anatomical fidelity and source verification	16 sources coded principally to this domain: 12 direct anatomy, 4 transferable HPE	Plausibility is not anatomical truth; performance varies by model, modality, version, and task.	Qualified verification of learner-facing/authoritative content and generated sources.	E/P	Most studies are bounded evaluations and should not be generalized across future systems.
4. Variation, bias, and representation	Seven sources coded principally to this domain: all transferable HPE	Demographic/phenotypic representation problems are documented; direct evidence concerning clinically relevant variants remains limited.	Review visual sets for representation; review important anatomical variants where educationally relevant.	E/P*	Empirical support is mainly transferable; representation and anatomical variation have different evidentiary bases.
5. Assessment validity and integrity	10 sources coded principally to this domain: 6 direct anatomy, 4 transferable HPE	GenAI may assist assessment, but satisfactory local psychometrics do not establish full validity or autonomous scoring safety.	Apply content/blueprint review, task validation, human approval, and appropriate post-use monitoring.	E/P	Autonomous consequential GenAI assessment remains insufficiently evaluated.
6. Provenance, intellectual property, and disclosure	Eight sources coded principally to this domain: three conceptual/ethical, three professional/normative, two direct anatomy	AI-assisted resources raise linked questions of provenance, permission, attribution, transformation, disclosure, and accountability.	Document material provenance/permissions; proportionately disclose GenAI involvement; identify accountable approval.	E/G/P	Legal implications vary by jurisdiction, contract, platform, and source material.
7. Human oversight and accountability	Six sources coded principally to this domain: four transferable HPE, two professional/normative	Supervision and accountability should vary by task and consequence.	Assign accountable owner; define permitted autonomy; retain challenge/override and human responsibility for consequential decisions.	E/G/P	Anatomy-specific implementation evidence is sparse.
8. Institutional authorization, monitoring, and incident response	Seven sources coded principally to this domain: four transferable HPE, three professional/normative	Authorization should remain conditional and lifecycle-based.	Maintain proportionate records, review triggers, monitoring, incident response, and revalidation.	E/G/P	Optimal monitoring frequency, burden, restart criteria, and outcome benefit remain unvalidated in anatomy.

The framework also builds on existing HPE scholarship concerning responsible AI and graduated supervision. Masters' AMEE Guide No. 158 provides an established ethical framework for AI use in HPE [10], while Gin et al. apply entrustment concepts to AI and emphasize graduated responsibility and supervision [11]. The present framework therefore does not claim novelty for graduated supervision, entrustment, or human accountability themselves. Its proposed contribution is to embed such oversight within an anatomy-specific architecture that also addresses donor-derived inputs, anatomical fidelity, variation and representation, assessment, provenance, and lifecycle governance.

A proposed risk-tiered framework for anatomy education

Risk Attaches to the Defined Use Case, Not the AI Product

The proposed unit of governance is the defined educational use case, not an AI product in the abstract. The same general-purpose model may be used for private brainstorming, development of a reusable learner handout, creation of an anatomical illustration, processing of donor-derived material, or contribution to summative assessment. Those uses do not present equivalent governance requirements.

Each defined use case is characterized according to six dimensions: purpose; audience; input and data class; consequence; scale; and permitted AI autonomy.

These dimensions form an author-developed, evidence-informed classification construct. They do not constitute a validated anatomy risk score or empirically established taxonomy.

Four Proposed Levels of Authorization and Control

Four tiers are proposed: Low, Moderate, High, and Restricted/Avoid. The highest triggered criterion determines the provisional tier as a conservative design rule. The rule is intended to prevent a high-consequence feature from being diluted by otherwise low-risk aspects of a workflow, but it is entirely author-proposed and may result in over-classification. Its inter-rater reliability, sensitivity to actual harm, and administrative burden require prospective evaluation. The four proposed risk tiers, their triggers, minimum controls, authorization requirements, monitoring and escalation provisions, and evidentiary status are summarized in Table 3.

**Table 3:** Proposed risk-tier authorization matrix for generative AI use in anatomy education The four tiers and their boundaries are author-developed and have not been empirically calibrated. The highest triggered criterion determines the provisional tier as a conservative design rule. Status codes are defined in the Table 2 legend. Local law, donor-program policy, institutional rules, assessment regulations, procurement requirements, and platform terms remain controlling. AI: artificial intelligence; QA: quality assurance; LMS: learning management system

Risk tier	Primary triggers and typical uses	Minimum proposed controls	Authorization and permitted AI role	Monitoring/escalation	Status
Low	Private, nonsensitive, nonconsequential support. No donor-derived/confidential/restricted input and no direct learner consequence. Examples: brainstorming, outlining, wording alternatives, nonconsequential administrative support.	Follow applicable institutional AI rules; educator reviews any material before it becomes educational content.	Individual educator within policy. AI may assist or draft.	Minimal documentation where locally required. Reclassify if material becomes learner-facing/reusable, restricted data are introduced, or consequence increases.	P
Moderate	Learner-facing or reusable material from nonsensitive inputs with low consequence. Routine course/cohort distribution and normal LMS placement may remain Moderate when properly reviewed.	Qualified anatomy review; source verification; structural fidelity checks; visual-set representation review; variation review where educationally relevant; proportionate disclosure.	Course/module lead or locally defined process. A0/A1 autonomy ordinarily appropriate; each learner-facing output approved before release.	Record responsible owner, use case, model/provider where known, verifier, limitations, and relevant approvals. Reassess after material change, recurrent error, restricted input, increased consequence, High scale, or High autonomy.	E/P
High	Authorized donor-derived/restricted processing; summative or otherwise consequential assessment; formal AI-generated anatomical exemplar; High scale; or High autonomy.	Task-specific validation; structured expert QA; donor/privacy/security review where applicable; assessment review/psychometrics where relevant; named accountability.	Department plus relevant assessment, donor, privacy/security, data-governance, or institutional authority. Strong supervision required.	Maintain formal use record and use-case-relevant monitoring. Reassess after material change, incident, recurrent error, or deterioration in validated performance.	E/G/P
Restricted/Avoid	Unauthorized restricted-input processing; A3 autonomous consequential learner decisions; autonomous/unverified release of generated anatomy as authoritative ground truth; use outside mandatory authorization conditions.	Correct the authorization, data, validation, or autonomy problem before proceeding; post-hoc checking alone is insufficient.	Do not proceed under ordinary authorization. Any exception requires appropriate institutional/legal/ethical authority.	Contain breaches; preserve relevant records; correct/withdraw outputs or decisions; investigate control failure; revalidate before any authorized restart.	E/G/P

Technical integration is not an independent classification dimension or High-tier trigger. It matters only when it changes an existing dimension, for example by introducing restricted inputs, increasing autonomy or scale, automatically releasing content, or linking GenAI directly to a consequential decision.

Operational Definitions of Key Threshold Terms

To support consistent classification, Table 4 defines the threshold terms used in the tiering framework and provides locally adjustable operational anchors.

**Table 4:** Proposed operational definitions of threshold terms Operational anchors are intended for local adaptation and are not validated cut-points. Status codes are defined in the Table 2 legend. HPE: health professions education; LMS: learning management system

Term	Proposed operational definition	Operational anchor	Status	Principal limitation
High scale	Deployment in which breadth, persistence, or volume materially increases potential error impact or makes effective oversight more difficult.	Examples include repeated reuse across multiple cohorts/programs without renewed review; interinstitutional/public dissemination; substantial-volume automated generation/release. Normal single-course/cohort or routine LMS use does not independently constitute a High scale.	P	Qualitative threshold is intentionally local and uncalibrated.
AI autonomy	Extent to which AI output reaches learners or affects educational processes without qualified human review of that specific instance before effect.	A0: assistive. A1: supervised generation with instance-level prerelease approval. A2: sampled/exception-based oversight; some outputs lack instance-level prerelease review. A3: autonomous action without instance-level prerelease review. A2/A3 = High autonomy. A3 is Restricted/Avoid when releasing authoritative anatomy or making consequential learner decisions.	E/G/P	A0-A3 ladder is author-developed but conceptually aligned with HPE supervision/entrustment; consequential A3 boundary is precautionary.
Material model or workflow change	Any change not covered by previous authorization/validation that could plausibly alter quality, safety, validity, or governance assumptions.	Provider/model/version, modality/tool access, retrieval corpus, system configuration, fine-tuning, data-handling terms, or changes to audience, inputs, consequence, scale, or autonomy.	E/P	Evidence supports version/task dependence; trigger definition is proposed.
Significant incident	Event materially breaching authorization conditions or causing/credibly risking consequential harm to learners, donor interests, privacy/confidentiality, assessment integrity, or another protected interest.	Examples: restricted data leaving scope; incorrect authoritative anatomy reaching learners; consequential assessment effect; material consent/privacy/security/permission breach. Error intercepted before effect may be treated as a near miss.	G/P	Operational incident boundary is proposed and informed by lifecycle-governance principles.
Qualified anatomical verification	Review by a person competent to teach, examine, or make expert judgments about the anatomy at the intended level, using independent authoritative sources.	Check identity/nomenclature; course/attachments/relations; branching/segmentation/count; laterality/orientation; spatial relationships; labels; relevant variation; generated references. Fluency, photorealism, model reputation, or memory alone are insufficient.	E/P	Need is supported by direct anatomical evidence; the specific checklist is proposed.

At authorization, each Moderate- or High-risk use should have a documented planned review date or review interval, together with defined events that trigger earlier reassessment. The interval should reflect consequence, persistence, model stability, and the institution's ability to detect material changes. No universal numerical interval is proposed because the available evidence does not establish an empirically justified frequency.

Five control gates and four proposed non-delegable human decisions

Tier assignment determines governance intensity but does not replace the substantive checks required by the use case. Before a Moderate- or High-risk use is released or operated, it should pass each applicable control gate. A failed gate should lead to correction, suspension, or escalation rather than reassignment to a lower tier.

Gate 1: Inputs, provenance, and platform suitability. Establish provenance, applicable permission or consent, relevant de-identification considerations, and platform authorization before donor-derived, confidential, identifiable, or otherwise restricted material enters a workflow.

Gate 2: Anatomical fidelity and source verification. Verify authoritative or learner-facing anatomy, including relevant structural relationships, labels, recognition, and generated references. The need for verification has substantial direct anatomy support; the exact proposed checklist is author-developed.

Gate 3: Variation and representation. Review demographic and phenotypic representation at image-set level where applicable. Where clinically relevant anatomical variation is part of the educational objective, assess important variants separately. Evidence for the representation component is mainly transferable; direct evidence for GenAI representation of anatomical variants remains limited.

Gate 4: Assessment validity and integrity. Where GenAI contributes to assessment, apply content and blueprint review, cognitive-level review where relevant, task-specific validation, quality and fairness checks, human approval, and proportionate post-use monitoring.

Gate 5: Disclosure and accountability. Document meaningful AI involvement proportionately, maintain appropriate provenance and permissions, identify an accountable human owner or approver, and preserve suitable routes for challenge, correction, or override.

Four decisions are proposed as non-delegable human decisions: (1) final acceptance of authoritative learner-facing anatomical claims, labels, images, or formal exemplars, (2) authorization to process donor-derived, confidential, identifiable, or otherwise restricted material, particularly beyond established permission, (3) final summative, progression, pass/fail, disciplinary, or contested consequential learner judgments, and (4) authorization to restart a suspended High-risk use following a significant incident. These are governance proposals informed to differing degrees by direct evidence, professional guidance, transferable evidence, and precautionary reasoning; they should not be described as empirically validated universal rules. Table 5 maps the five pressure points and major framework components to their primary governance domains, the nature of the supporting evidence, evidentiary status, and principal limitations.

**Table 5:** Framework-to-evidence crosswalk The crosswalk distinguishes documented failure modes and professional principles from operational rules proposed in this framework. The four tiers, highest-trigger rule, A0-A3 autonomy ladder, control-gate procedures, and four proposed non-delegable decisions have not been prospectively validated. Status codes are defined in the Table 2 legend. HPE: health professions education; AI: artificial intelligence

Framework component	Operational statement	Primary domain(s)	Nature of evidence	Status	Principal limitation
Donor pressure point	Donor material retains provenance, consent/permission, dignity, privacy, and stewardship obligations after digitization.	2	Professional/ethical + limited direct anatomy	G/P	Operational controls partly proposed.
Fidelity pressure point	Generated anatomy requires structural and source-level verification.	3	Strong direct anatomy evidence	E/P	Failure mode documented; exact verification procedure proposed.
Variation/representation pressure point	Anatomical variation and demographic/phenotypic representation are distinct but related concerns.	4	Representation evidence transferable; variation evidence sparse	E/P	Variation safeguard precautionary.
Assessment pressure point	Governance intensity rises when AI output contributes to consequential assessment.	5	Direct anatomy + transferable HPE	E/P	Exact nondelegation threshold precautionary.
Provenance/disclosure pressure point	Origin, transformation, permission, AI involvement, and accountability should remain traceable.	6	Direct + professional + conceptual/ethical	E/G/P	Legal requirements jurisdiction-dependent.
Use-case classification	Classify purpose, audience, input/data class, consequence, scale, and autonomy.	1 + cross-cutting	Predominantly transferable/normative	P	Author-developed and unvalidated.
Human oversight	Assign human ownership and match supervision to consequence/autonomy.	7	Transferable HPE + professional	E/G/P	Direct anatomy implementation evidence limited.
Lifecycle governance	Authorization, monitoring, incident response, correction, and revalidation continue after initial approval.	8	Transferable HPE + professional	E/G/P	Effectiveness/burden unvalidated in anatomy.
Highest-trigger rule	Highest triggered criterion sets provisional tier.	1, 7, 8	Conservative author design	P	Not calibrated; possible over-classification.
Low tier	Private nonsensitive nonconsequential support remains educator-managed.	1, 7, 8	Normative/transferable	P	Boundary proposed.
Moderate tier	Reusable/learner-facing nonsensitive low-consequence content requires prerelease qualified review.	3, 4, 6, 7	Direct + transferable	E/P	Tier boundary proposed.
High tier	Donor/restricted processing, consequential assessment, formal anatomical exemplars, High scale, or High autonomy trigger stronger governance.	2, 3, 5, 7, 8	Mixed empirical/professional/transferable	E/G/P	Tier boundary proposed.
Restricted/Avoid tier	Unauthorized restricted processing, autonomous consequential decisions, or autonomous/unverified authoritative anatomy should not proceed ordinarily.	2, 3, 5, 7, 8	Normative/precautionary + empirical rationale	E/G/P	Not a universal legal prohibition.
Gate 1	Confirm provenance, permission, identifying features, and platform suitability.	2, 6	Professional/ethical	G/P	Principle guidance-supported; details proposed.
Gate 2	Verify structural anatomy, claims, images, labels, and references.	3	Direct anatomy	E/P	Empirical rationale; checklist proposed.
Gate 3	Review representation and relevant anatomical variation.	4	Mainly transferable	E/P	Variation component remains under-evidenced.
Gate 4	Apply assessment content/blueprint review, validation, human approval, and monitoring.	5	Direct + transferable	E/P	Exact workflow proposed.
Gate 5	Document AI contribution and named accountability; preserve challenge/override.	6, 7, 8	Professional/normative + transferable	E/G/P	Implementation context-dependent.
Proposed human decision 1	Final authoritative anatomy acceptance remains human.	3, 7	Empirical rationale + governance	E/P	Absolute rule proposed.
Proposed human decision 2	Restricted-input authorization remains human/institutional.	2, 7	Professional/ethical	G/P	Scope depends on consent/law/policy.
Proposed human decision 3	Final consequential learner judgments remain human-accountable.	5, 7	GenAI limitations + transferable assessment + governance	E/G/P	Precautionary boundary; not directly validated.
Proposed human decision 4	Restart after significant High-risk incident requires governance approval.	7, 8	Lifecycle governance	G/P	Restart criteria unvalidated.
Lifecycle loop	Govern inputs -> verify -> validate where applicable -> deploy -> monitor -> respond -> revalidate/reclassify.	2-8	Cross-cutting synthesis	E/G/P	Frequency, burden, and outcome benefit unknown.

Reclassification, monitoring, incident response, and revalidation

Authorization is conditional and revisable rather than permanent. Reassessment should occur when one or more characteristics supporting the original tier or authorization materially change.

Potential triggers include introduction of donor-derived, confidential, identifiable, or otherwise restricted inputs; change in purpose or audience; transition from formative to summative use; material increase in scale; increased AI autonomy; changes in provider, model, modality, retrieval system, workflow, or data-handling conditions; recurrent material errors or near misses; deterioration of assessment performance where relevant; new relevant safety evidence; or a significant incident.

A material change does not automatically require prohibition or elevation to High risk. The affected characteristics and controls should be reassessed and the tier, validation requirements, and authorization reconsidered accordingly.

Monitoring should be proportionate to the actual use case. Moderate- and High-risk uses should ordinarily maintain enough information to reconstruct the approved use, accountable owner, relevant provider/model where known, tier, applicable gates, authorization, verification or validation, important limitations, material changes, incidents or near misses, and corrective action.

Monitoring should focus on relevant failure modes rather than generic surveillance. Depending on the use, these may include anatomical errors, reference failures, assessment performance problems, unauthorized exposure of restricted material, recurrent representational problems, or unexpected changes after model or workflow modification.

Scheduled review can complement but should not replace event-triggered review. Where providers do not make model updates sufficiently transparent, programs should use the locally documented review interval established at authorization, but no universal calendar interval is prescribed.

Incident response should also be proportionate. Where continuing operation may create material risk, actions may include temporary containment or suspension, preservation of relevant records, assessment of affected learners or donor interests, correction or withdrawal of affected material or decisions, review of the failed or absent control, modification of the workflow, and targeted revalidation.

An error intercepted before release may be treated as a near miss where documenting it provides useful information about control performance. Routine editing corrections need not automatically become formal incidents.

Revalidation should address what changed or failed rather than requiring repetition of every control automatically. A model change affecting anatomical images may require renewed fidelity testing, whereas a change in data-retention terms may instead require renewed privacy or donor-governance review. If a significant incident results in suspension of a High-risk use, restart should require documented approval by the responsible governance authority. Figure 1 presents the resulting anatomy-specific GenAI governance decision algorithm. Figure 2 summarizes the conditional lifecycle of authorization, input governance, output verification, assessment validation where applicable, deployment, monitoring, incident response, and revalidation or reclassification.

**Figure 1:**
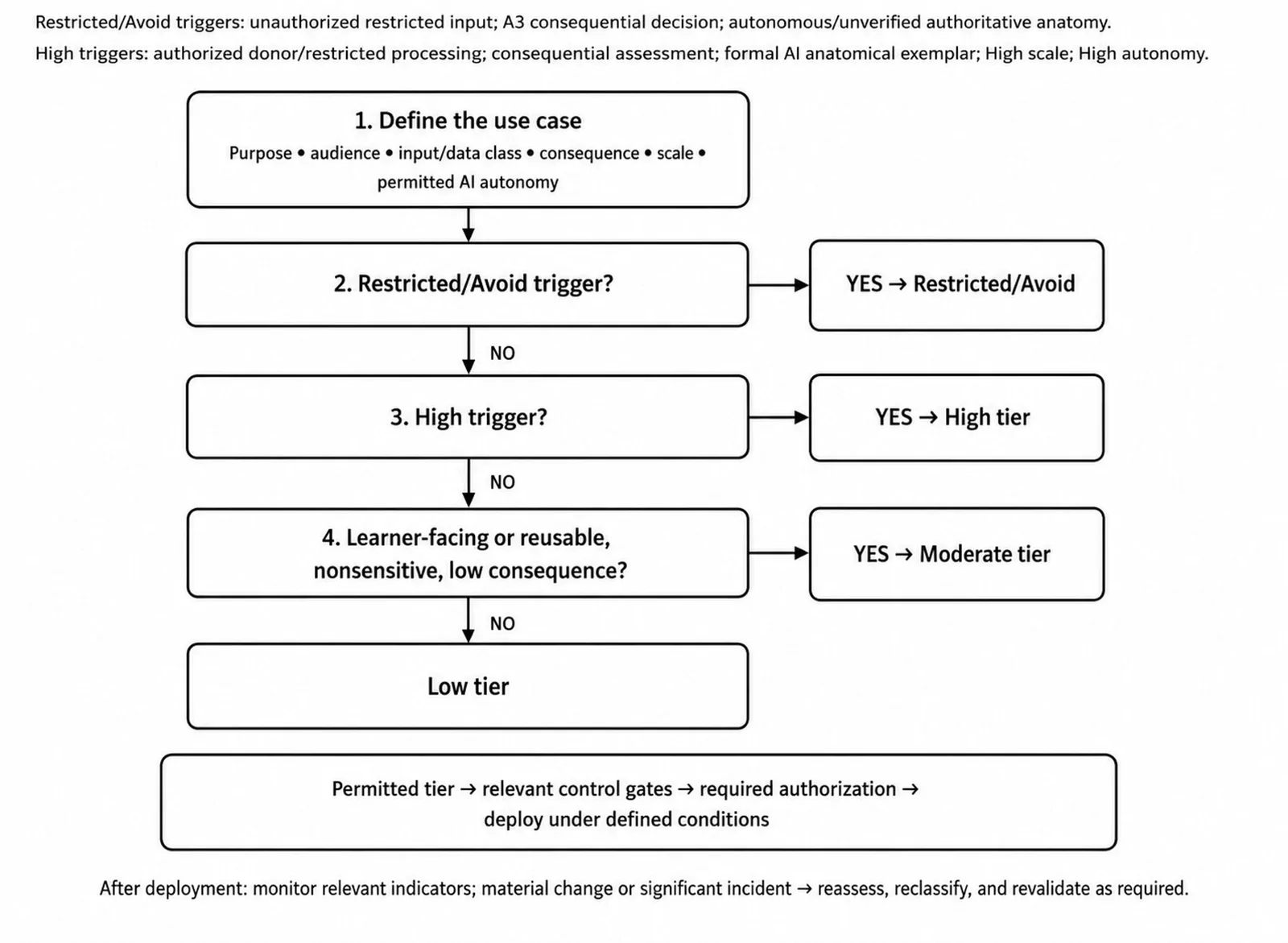
Anatomy-specific generative artificial intelligence governance decision algorithm The defined educational use case is characterized by purpose, audience, input/data class, consequence, scale, and permitted AI autonomy. Restricted/Avoid triggers are considered first, followed by High-tier triggers; remaining learner-facing or reusable low-consequence uses are classified as Moderate, while private, nonsensitive, nonconsequential uses may remain Low. The highest triggered criterion determines the provisional tier. Relevant control gates, named human or institutional authorization, proportionate monitoring, incident response, and revalidation follow. The algorithm is an author-developed, evidence-informed proposal and has not been empirically calibrated or prospectively validated. Original schematic prepared using Matplotlib (version 3.10.8; Matplotlib Development Team, via NumFOCUS, Inc., Austin, TX, USA).

**Figure 2:**
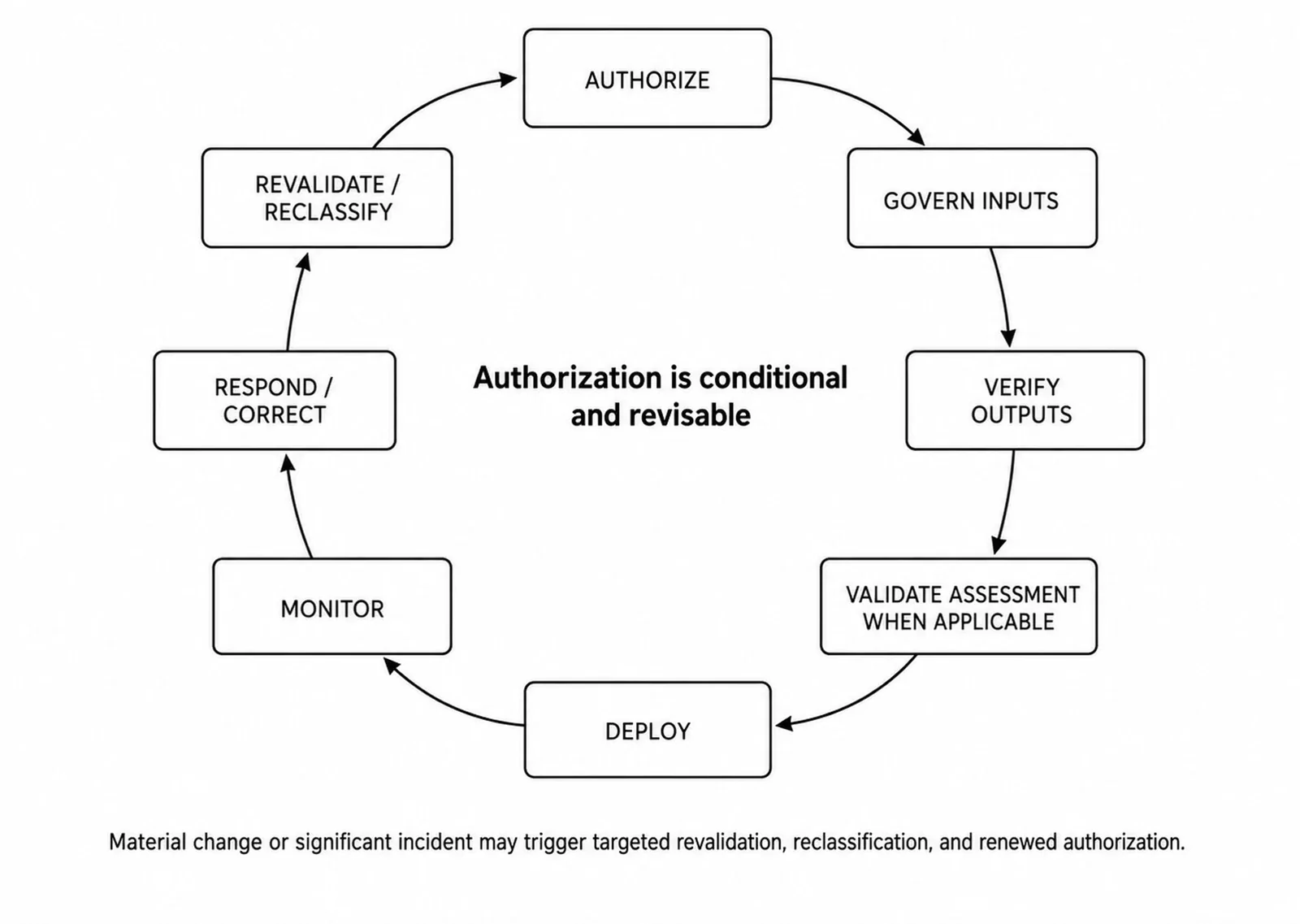
Anatomy-AI governance lifecycle control loop Authorization is conditional and continuing rather than permanent. Approved uses remain subject to applicable input governance, output verification, assessment validation where relevant, proportionate monitoring, incident response, correction or withdrawal, and revalidation. A material change or significant incident may trigger reclassification and renewed authorization. No universal calendar-based revalidation interval is prescribed because the optimal review frequency for anatomy-education GenAI use has not been empirically established. Original schematic prepared using Matplotlib (version 3.10.8; Matplotlib Development Team, via NumFOCUS, Inc., Austin, TX, USA). GenAI: generative artificial intelligence

Regulatory context: partial convergence with the EU AI Act

The proposed framework was not derived from a regulatory classification system. Nevertheless, the use of consequence as a reason for heightened governance shows partial convergence with the European Union Artificial Intelligence Act.

Annex III of Regulation (EU) 2024/1689 identifies several education-related AI systems as high-risk, including systems intended to determine access or admission, evaluate learning outcomes, assess the appropriate level of education a person receives or may access, and monitor or detect prohibited behavior during tests [34]. The statutory classification is subject to the Act's qualifications and should not be generalized to every educational AI use. The 2026 amending regulation did not remove education and vocational-training systems from the Annex III classification list.

This provides relevant external regulatory context for placing consequential assessment under heightened control, but it does not validate the proposed High tier. The EU Act regulates AI systems according to statutory criteria, whereas the present framework classifies defined educational use cases by purpose, audience, input/data class, consequence, scale, and autonomy. The two classification systems should therefore not be presented as equivalent.

Regulation (EU) 2026/1744 amended the application timetable. Under amended Article 113, Chapter III, Sections 1-3, with the exception specified in the Regulation, apply from December 2, 2027, for systems classified as high-risk pursuant to Article 6(2) and Annex III and from August 2, 2028, for systems classified pursuant to Article 6(1) and Annex I [35]. Regulation (EU) 2026/1744 was published in the Official Journal on July 24, 2026, and entered into force on July 27, 2026.

Article 50 should be considered separately. It establishes role- and use-specific transparency obligations rather than a universal disclosure requirement applying identically to every learner-facing educational GenAI use. The regulation's general application date is August 2, 2026, while Regulation (EU) 2026/1744 introduced a specific transition for providers of synthetic-content systems already placed on the market before that date: those providers must comply with Article 50(2) by December 2, 2026 [35]. Institutions must therefore determine applicable obligations according to their role, the AI system, intended purpose, type of generated content, and jurisdiction.

The convergence is consequently limited but relevant. EU law independently recognizes that certain AI uses affecting educational access, learning outcomes, and examination processes can warrant heightened governance. It does not resolve the anatomy-specific problems addressed by this framework, including donor-derived inputs and qualified verification of anatomical content. The proposed framework is not a legal-compliance instrument.

Putting the framework into practice

Implementation should be proportionate and should reuse existing educational and institutional structures wherever possible. The framework is not intended to create a new committee or extensive approval process for every interaction with GenAI.

A minimum viable implementation consists of a local inventory of material governed uses, named accountability, use-case classification and tier-matched authorization, concise documentation of applicable controls, and defined escalation, incident-response, and revalidation pathways.

Accountability should attach to the use case rather than merely the approved tool. Routine learner-facing content may be owned by a course or module lead; assessment use may fall within an existing assessment committee; donor-derived processing may require anatomy or body-donation authority; and restricted data or external systems may require privacy, information-security, procurement, legal, compliance, or institutional AI-governance involvement.

The purpose of the use record is accountability rather than exhaustive surveillance. It should contain enough information to reconstruct why a material governed use was permitted and what safeguards applied. Exhaustive prompt archiving is not required by this framework.

Lower-risk activity should ordinarily proceed under standing institutional rules rather than universal case-by-case preapproval. Escalation should occur when a use introduces restricted inputs, becomes consequential, reaches the High-scale threshold, moves to High autonomy, creates unresolved consent or permission issues, or experiences a material change or significant incident.

Technical integration requires escalation only when it changes another risk dimension. For example, putting a reviewed handout into an LMS does not independently create High risk; connecting a GenAI workflow directly to a grading system so that outputs affect scores without instance-level human review changes consequence and autonomy and therefore does.

Analyses of institutional GenAI guidance also suggest why a discipline-specific operational layer may be useful. McDonald et al. found substantial variation in institutional guidance and cautioned that faculty-facing requirements can become burdensome [36], while Jin et al. identified persistent needs for clearer institutional roles, communication, and implementation frameworks [37]. The purpose of the anatomy-specific layer is therefore to reduce local ambiguity while avoiding unnecessary duplication of existing governance.

Existing course leadership, assessment committees, donor-program governance, educational-technology teams, privacy and security offices, procurement processes, ethics or compliance mechanisms, and institutional AI-governance structures should be reused where they already address the relevant control.

The proposed implementation model is intended to make governance proportionate rather than unnecessarily burdensome, but that benefit remains untested. Administrative workload, classification consistency, feasibility across differently resourced programs, and effects on educational or safety outcomes require prospective evaluation.

Applying the framework to this article

A framework that emphasizes disclosure, verification, and human accountability should make its own use of GenAI transparent.

Under the proposed framework, this manuscript-preparation workflow is classified as Moderate risk because it produced reusable scholarly material from published or publicly accessible information, did not require donor-derived, learner-specific, or confidential inputs, and did not itself make a consequential decision concerning a learner.

The authors retained responsibility for source selection and interpretation, framework decisions, manuscript content, and conclusions. GenAI was used for language checking rather than as an author, evidentiary source, or autonomous scholarly decision-maker. A detailed artificial intelligence disclosure is provided in the Acknowledgments.

Limitations

Several limitations constrain both the evidence synthesis and the proposed framework.

First, this was a structured, evidence-informed state-of-the-art review with framework development rather than a systematic or scoping review. Although six search streams have documented purposes, concept blocks, dates, and discovery or verification sources, exact historical query strings and complete retrieval, deduplication, screening, and exclusion counts were not prospectively archived. Those data have not been reconstructed retrospectively. The retained evidence and stream-level process are therefore auditable, but the historical search cannot be reproduced as a systematic search.

Second, structured bibliographic searching was limited primarily to PubMed/MEDLINE, supplemented by Google Scholar and targeted professional, institutional, publisher, and organizational sources. A broader prospectively defined set of databases such as Scopus, Web of Science, Embase, ERIC, IEEE Xplore, or ACM Digital Library was not searched. Relevant literature may therefore have been missed, particularly across education, computer science, ethics, law, policy, and rapidly emerging or non-indexed sources.

Third, no single formal risk-of-bias or certainty-of-evidence instrument was applied across the heterogeneous retained evidence. Descriptive appraisal and evidence classification were used instead. This limits standardized comparison of methodological quality. Preregistration status was not systematically captured or verified and was not used as an appraisal criterion.

Fourth, screening and source coding were performed by one author (AM), without independent double-screening, duplicate coding, or formal inter-rater reliability assessment. The domain and evidence-basis classifications therefore represent a transparent single-rater synthesis. Historical working relevance, credibility, and framework-utility fields are not used as evidence weights or certainty ratings because their exact historical operational role cannot now be reconstructed confidently.

Fifth, the evidence base is heterogeneous and unevenly distributed. Direct anatomy evidence is concentrated in anatomical fidelity and assessment. Donor governance relies mainly on professional guidance and ethical scholarship, whereas the evidence for representation, human oversight, and lifecycle governance is drawn predominantly from transferable HPE and professional sources. Many anatomy studies evaluate specific models, prompts, outputs, institutions, or time points rather than governance interventions. Their results should not be generalized automatically across models, versions, modalities, tasks, institutions, or future releases.

Sixth, important direct evidence gaps remain. Evidence concerning GenAI representation of clinically important anatomical variants, long-term representational effects, implementation of human-accountability models, anatomy-specific incident-response systems, and autonomous consequential GenAI assessment remains limited. Several proposed safeguards therefore rest on transferable evidence, professional guidance, ethical reasoning, or precautionary governance.

Seventh, the framework itself is author-developed and unvalidated. The six classification dimensions, four tiers, A0-A3 autonomy ladder, highest-trigger rule, operational definitions, five control gates, and four proposed non-delegable human decisions have not been prospectively tested as an integrated governance instrument. Tier boundaries are not empirically calibrated; inter-rater reliability is unknown; over- and under-classification have not been measured; and administrative burden, feasibility, educational impact, and reduction of governance failures remain unquantified.

Eighth, the framework addresses primarily educator- and institution-authorized workflows. Independent learner use of consumer GenAI outside such workflows is not operationally classified by the proposed tiers. Learner upload or transformation of donor-derived or otherwise restricted material nevertheless remains an important institutional-policy and research concern.

Finally, donor law, privacy requirements, intellectual property rules, assessment regulation, procurement requirements, contracts, platform terms, and AI regulation vary across jurisdictions and evolve over time. This framework is not a legal-compliance instrument and does not replace applicable law, donor agreements, institutional policy, professional standards, contractual requirements, or regulatory classifications.

Future research should therefore test whether independent educators classify identical use cases consistently; quantify administrative burden and feasibility; evaluate whether the proposed gates detect or prevent their targeted failures; examine the appropriateness of the highest-trigger and autonomy rules; and determine whether tier-matched governance measurably changes the quality, safety, fairness, or educational value of learner-facing material. The complete condensed source-level coding for the 71 retained substantive sources is presented in Table 6.

**Table 6:** Condensed evidence matrix of the 71 retained sources The final retained evidence set comprises 71 substantive sources. The Ref. column gives the formal manuscript reference number. Evidence-basis categories describe the role and disciplinary setting of each source: direct anatomy evidence; transferable HPE evidence; professional/normative guidance; or conceptual/ethical scholarship. “Direct anatomy evidence” does not imply that the AI modality was necessarily generative; Bernard et al. and Mezil et al. concern non-generative/conventional AI and are used only where their findings are transferable to governance questions such as automated assessment and human oversight. Historical working fields for relevance, source credibility, and framework utility are retained in the audit file, but their precise operational role cannot now be reconstructed with sufficient confidence; they are therefore not used in this revised article as inclusion/exclusion criteria, evidence weights, certainty ratings, or determinants of recommendation strength. Zilundu et al. [4] is cited in the Introduction as motivating contextual evidence concerning faculty-reported governance needs and was not included in the 71-source coded framework-development set. Methodological review-design references [12,13] and EU regulatory instruments [34,35] are likewise outside the retained evidence set and are used only for methodological or contemporary legal context. HPE: health professions education; EU: European Union; AI: artificial intelligence

Ref.	Source (first authors, year)	Source type	Evidence basis	Principal governance domain
[2]	Lazarus et al. (2024)	Critical review	Conceptual/ethical scholarship	Cross-cutting/multiple
[3]	Cornwall et al. (2024)	Ethics analysis	Conceptual/ethical scholarship	2. Input and donor-derived material governance
[1]	Yi et al. (2026)	Critical review	Direct anatomy evidence	Cross-cutting/multiple
[5]	Sonnenberg et al. (2026)	Consensus statement	Transferable HPE evidence	Cross-cutting/multiple
[14]	International Federation of Associations of Anatomists (2023)	Professional guideline	Professional/normative guidance	2. Input and donor-derived material governance
[15]	Jones et al. (2026)	Professional guideline	Professional/normative guidance	Cross-cutting/multiple
[17]	Cornwall et al. (2016)	Ethics analysis	Conceptual/ethical scholarship	2. Input and donor-derived material governance
[16]	Iwanaga et al. (2025)	Editorial/perspective	Conceptual/ethical scholarship	6. Provenance, IP and disclosure
[19]	Eldesoqui et al. (2025)	Original empirical research	Direct anatomy evidence	3. Anatomical fidelity and source verification
[26]	Alon et al. (2026)	Systematic review	Transferable HPE evidence	4. Variation, bias and representation
[38]	Gupta et al. (2026)	Scoping review	Transferable HPE evidence	3. Anatomical fidelity and source verification
[39]	Agarwal et al. (2026)	Scoping review	Transferable HPE evidence	5. Assessment validity and integrity
[40]	Che and Liu (2026)	Narrative review	Transferable HPE evidence	3. Anatomical fidelity and source verification
[41]	World Health Organization (2024)	Professional guideline	Professional/normative guidance	7. Human oversight and accountability
[42]	Abdellatif et al. (2022)	Narrative review	Conceptual/ethical scholarship	Cross-cutting/multiple
[43]	Joseph et al. (2025)	Systematic review	Direct anatomy evidence	Cross-cutting/multiple
[44]	Chytas et al. (2025)	Scoping review	Direct anatomy evidence	3. Anatomical fidelity and source verification
[45]	Erbek et al. (2026)	Systematic review	Direct anatomy evidence	3. Anatomical fidelity and source verification
[46]	Collins et al. (2024)	Original empirical research	Direct anatomy evidence	1. Use-case and risk classification
[47]	Temizsoy Korkmaz et al. (2025)	Original empirical research	Direct anatomy evidence	3. Anatomical fidelity and source verification
[48]	Topcu et al. (2026)	Original empirical research	Direct anatomy evidence	2. Input and donor-derived material governance
[49]	Morgan et al. (2026)	Other	Direct anatomy evidence	5. Assessment validity and integrity
[50]	Almamari et al. (2026)	Original empirical research	Direct anatomy evidence	Cross-cutting/multiple
[51]	Bolgova and Mavrych (2025)	Original empirical research	Direct anatomy evidence	3. Anatomical fidelity and source verification
[52]	Hennessy et al. (2020)	Professional guideline	Professional/normative guidance	6. Provenance, IP and disclosure
[18]	Human Tissue Authority (2023)	Professional guideline	Professional/normative guidance	2. Input and donor-derived material governance
[53]	Human Tissue Authority (2025)	Policy/framework document	Professional/normative guidance	2. Input and donor-derived material governance
[54]	Passalacqua et al. (2025)	Consensus statement	Professional/normative guidance	2. Input and donor-derived material governance
[55]	Keet and Kramer (2022)	Ethics analysis	Conceptual/ethical scholarship	Cross-cutting/multiple
[56]	Lottering et al. (2022)	Original empirical research	Direct anatomy evidence	6. Provenance, IP and disclosure
[33]	Cornwall et al. (2025)	Ethics analysis	Conceptual/ethical scholarship	6. Provenance, IP and disclosure
[32]	Ho et al. (2026)	Original empirical research	Direct anatomy evidence	6. Provenance, IP and disclosure
[57]	Rossetti et al. (2026)	Systematic review	Conceptual/ethical scholarship	6. Provenance, IP and disclosure
[58]	Kramer and Moxham (2026)	Editorial/perspective	Conceptual/ethical scholarship	2. Input & donor-derived material governance
[59]	Iwanaga et al. (2022)	Consensus statement	Professional/normative guidance	6. Provenance, IP and disclosure
[20]	Hyeamang et al. (2025)	Original empirical research	Direct anatomy evidence	3. Anatomical fidelity and source verification
[21]	Mavrych et al. (2025)	Original empirical research	Direct anatomy evidence	3. Anatomical fidelity and source verification
[60]	Al-Khater (2025)	Original empirical research	Direct anatomy evidence	3. Anatomical fidelity and source verification
[61]	Sivri et al. (2026)	Original empirical research	Direct anatomy evidence	3. Anatomical fidelity and source verification
[25]	Paslı et al. (2026)	Original empirical research	Direct anatomy evidence	3. Anatomical fidelity and source verification
[23]	Ülkir and Paslı (2026)	Original empirical research	Direct anatomy evidence	3. Anatomical fidelity and source verification
[24]	Sarantopoulos et al. (2026)	Original empirical research	Transferable HPE evidence	3. Anatomical fidelity and source verification
[22]	Bae et al. (2025)	Original empirical research	Direct anatomy evidence	3. Anatomical fidelity and source verification
[62]	Kumar et al. (2024)	Original empirical research	Transferable HPE evidence	3. Anatomical fidelity and source verification
[63]	Currie et al. (2024)	Original empirical research	Transferable HPE evidence	4. Variation, bias and representation
[64]	Bui et al. (2026)	Original empirical research	Transferable HPE evidence	4. Variation, bias and representation
[65]	Shaari et al. (2025)	Original empirical research	Transferable HPE evidence	4. Variation, bias and representation
[66]	Currie et al. (2025)	Original empirical research	Transferable HPE evidence	4. Variation, bias and representation
[67]	Ali et al. (2024)	Original empirical research	Transferable HPE evidence	4. Variation, bias and representation
[68]	Senft Everson et al. (2026)	Original empirical research	Transferable HPE evidence	4. Variation, bias and representation
[28]	Lopiano et al. (2026)	Original empirical research	Direct anatomy evidence	5. Assessment validity and integrity
[27]	Kıyak et al. (2025)	Original empirical research	Direct anatomy evidence	5. Assessment validity and integrity
[29]	Tekin et al. (2025)	Original empirical research	Direct anatomy evidence	5. Assessment validity and integrity
[31]	Bernard et al. (2024)	Original empirical research (non-generative/conventional AI: decision-tree automated grading)	Direct anatomy evidence; transferable to GenAI governance	5. Assessment validity and integrity
[69]	Mezil et al. (2026)	Original empirical research (non-generative/conventional AI)	Direct anatomy evidence; transferable to GenAI governance where applicable	5. Assessment validity and integrity
[70]	Al-Mashhadani et al. (2026)	Systematic review	Transferable HPE evidence	5. Assessment validity and integrity
[71]	Ahmed et al. (2025)	Original empirical research	Transferable HPE evidence	5. Assessment validity and integrity
[30]	Meo et al. (2024)	Original empirical research	Transferable HPE evidence	5. Assessment validity and integrity
[72]	Association of American Medical Colleges (2025)	Professional guideline	Professional/normative guidance	8. Institutional authorization, monitoring and incident response
[10]	Masters (2023)	Professional guideline	Transferable HPE evidence	7. Human oversight and accountability
[6]	Autio et al. (2024)	Policy/framework document	Professional/normative guidance	8. Institutional authorization, monitoring and incident response
[7]	Lekadir et al. (2025)	Consensus statement	Transferable HPE evidence	8. Institutional authorization, monitoring and incident response
[11]	Gin et al. (2025)	Policy/framework document	Transferable HPE evidence	7. Human oversight and accountability
[73]	Izquierdo-Condoy et al. (2026)	Narrative review	Transferable HPE evidence	8. Institutional authorization, monitoring and incident response
[9]	Organisation for Economic Co-operation and Development (2024)	Policy/framework document	Professional/normative guidance	7. Human oversight and accountability
[8]	Miao and Holmes (2023)	Professional guideline	Professional/normative guidance	8. Institutional authorization, monitoring and incident response
[37]	Jin et al. (2025)	Original empirical research	Transferable HPE evidence	7. Human oversight and accountability
[74]	Ganguly et al. (2025)	Original empirical research	Transferable HPE evidence	7. Human oversight and accountability
[36]	McDonald et al. (2025)	Original empirical research	Transferable HPE evidence	8. Institutional authorization, monitoring and incident response
[75]	International Committee of Medical Journal Editors (2026)	Professional guideline	Professional/normative guidance	6. Provenance, IP and disclosure
[76]	Alfredo et al. (2024)	Systematic review	Transferable HPE evidence	8. Institutional authorization, monitoring and incident response

## Conclusions

Anatomy education needs an operational layer between general responsible AI principles and daily practice. This article proposes classifying the defined use case rather than the AI product. The highest triggered criterion sets a provisional tier, with stronger controls for donor-derived inputs, authoritative learner-facing anatomy, and consequential assessment, while human accountability is retained. Authorization should remain conditional on monitoring, incident response, and revalidation.

The framework is an evidence-informed proposal for local adaptation, not a validated instrument or legal-compliance tool. Prospective evaluation should test classification consistency, feasibility, administrative burden, and effects on educational quality and safety. Applicable law, donor-program policy, assessment rules, and institutional governance remain controlling.

## Appendices

**Table 7:** Source-level evidence matrix for the 71 retained substantive sources This appendix contains the complete source-level coding for the 71 substantive sources retained in the evidence synthesis. Evidence-basis categories are direct anatomy evidence, transferable health professions education (HPE) evidence, professional or normative guidance, and conceptual or ethical scholarship. “Anatomy-context source?” indicates whether a source concerns anatomy, anatomical material, anatomy education, or anatomy-related governance and is distinct from the narrower “direct anatomy evidence” category. AI modality is classified as GenAI, conventional AI (foundational/transferable), general AI/mixed modalities, or not applicable (non-AI source). “Illustrative tier relevance” is a historical working field and does not classify the source itself. Relevance, source credibility, framework utility, and triage-priority fields are retained as historical working fields; their precise operational role cannot now be reconstructed and they are not used in the revised manuscript as eligibility criteria, evidence weights, certainty ratings, recommendation-strength measures, or determinants of framework inclusion. This appendix contains retained sources only; historical record-level exclusion data were not preserved and are not retrospectively reconstructed. Principal governance-domain coding corresponds to the eight domains presented in main-text Table 2. DOI or stable URL fields are provided where available.

ID	Citation key	Full citation	Year	Country/region	DOI/URL	Source type	Publication status	Peer reviewed?	Discipline/context	Anatomy-context source?	Evidence basis	AI modality	Educational use	Input/data type	Governance domain (Provisional)	Governance problem	Ethical concern	Potential harm	Primary stakeholder	Key finding/evidence	Proposed safeguard/control	Responsible actor	Donor relevance	Assessment relevance	Verification relevance	Illustrative tier relevance (historical working field)	Relevance to review (1-5)	Source credibility (1-5)	Framework utility (1-5)	Triage priority	Key evidence locator/descriptive summary	Manuscript use	Notes
1	Lazarus, 2024	Lazarus MD, Truong M, Douglas P, Selwyn N. Artificial intelligence and clinical anatomical education: Promises and perils. Anatomical Sciences Education. 2024;17(2):249-262. doi:10.1002/ase.2221.	2024	Australia	https://doi.org/10.1002/ase.2221	Critical review	Published	Yes	Anatomy education	Yes	Conceptual/ethical scholarship	Conventional AI (foundational only)	Learning support	No sensitive input	Cross-cutting/multiple	AI adoption may fail to represent uncertainty, human variation, diversity and learner needs.	Equity, transparency, preservation of human-centered learning, and appropriate limits on AI.	Misrepresentation of anatomy/variation, inequity, over-reliance, and erosion of educational benefits.	Multiple stakeholders	Identifies five tensions in anatomy AI adoption: human variation, healthcare practice, diversity/social justice, student support, and student learning; proposes a considered rather than wholesale adoption approach.	Use transparency; include uncertainty and anatomical variation; develop educator awareness of AI limitations; preserve AI-free learning where educationally justified; use AI to extend rather than replace human capacity.	Individual educator	No	Partial/indirect	Partial/indirect	Moderate	5	5	5	5	Abstract; five tensions and practical recommendations.	Competitor/novelty analysis	Anchor source; major anatomy-specific conceptual competitor.
2	Cornwall, 2024	Cornwall J, Hildebrandt S, Champney TH, Goodman K. Ethical concerns surrounding artificial intelligence in anatomy education: Should AI human body simulations replace donors in the dissection room? Anatomical Sciences Education. 2024;17(5):937-943. doi:10.1002/ase.2335.	2024	New Zealand/United States	https://doi.org/10.1002/ase.2335	Ethics analysis	Published	Yes	Anatomy education/body donation ethics	Yes	Conceptual/ethical scholarship	Conventional AI (foundational only)	Learning support	Cadaveric/donor image	2. Input & donor-derived material governance	Whether AI human-body simulations should replace donor-based anatomy learning.	Respect for donors, bioethics, professional identity formation, and preservation of uniquely human educational experiences.	Loss of nontechnical, ethical and professional-learning benefits associated with donor exposure.	Learner	Argues that the known educational and professional-formation benefits of donor-based dissection currently outweigh the uncertain benefits of replacing donors with AI simulations.	Protect donor interaction rather than replace it with AI simulations without strong evidence; scrutinize substitution decisions.	Anatomy department	Yes	No	Partial/indirect	High	5	5	4	4.7	PubMed abstract; conclusions on preserving donor interaction.	Donor-derived materials	Anchor source; direct anatomy ethics competitor.
3	Yi, 2026	Yi KH, Wan J, Rosellini I, Lau KH, Lee W, Haykal D. Artificial Intelligence in Anatomic Education: Educational Utility, Safety Boundaries, and Implementation Considerations. Journal of Craniofacial Surgery. 2026 Mar 17. Online ahead of print. doi:10.1097/SCS.0000000000012573.	2026	International	https://doi.org/10.1097/SCS.0000000000012573	Critical review	Online first	Yes	Anatomic education	Yes	Direct anatomy evidence	General AI/mixed modalities	Learning support	No sensitive input	Cross-cutting/multiple	AI tools in anatomy may introduce accuracy, misinformation, hallucination, bias, infrastructure and accountability problems.	Educational safety, fairness, validation, accountability and appropriate human oversight.	Incorrect or biased anatomical learning resources and inadequate trainee preparation.	Multiple stakeholders	Critical synthesis concludes AI may complement anatomy-led curricula when anatomical accuracy is validated and use is accompanied by safeguards, human oversight and governance.	Validate anatomical accuracy; maintain human oversight; use governance safeguards; treat AI as complementary rather than autonomous.	Anatomy department	Partial/indirect	Partial/indirect	Yes	Moderate	5	4	5	4.7	PubMed abstract, lines describing accuracy validation, hallucination, bias, safeguards and governance.	Competitor/novelty analysis	Anchor source; strongest 2026 anatomy-specific review competitor.
4	Sonnenberg, 2026	Sonnenberg LK, Wiljer D, Mamdani M, et al. Principles for Responsible AI in Health Professions Education, Research, and Care: Health CARE-AI (Contextual, Accountable, Responsible, and Equitable Artificial Intelligence) Framework Delphi Consensus Study. JMIR Medical Education. 2026;12:e91626. doi:10.2196/91626.	2026	International	https://doi.org/10.2196/91626	Consensus statement	Published	Yes	Health professions education/health care	No	Transferable HPE evidence	General generative AI	Institutional governance	No sensitive input	Cross-cutting/multiple	Broad AI principles often lack practice-level direction for educators, learners and institutions.	Accountability, integrity, AI literacy, equity, transparency and collective responsibility.	Unaccountable or inequitable AI use across educational and professional settings.	Multiple stakeholders	A 3-phase modified Delphi produced 10 consensus principles for contextual, accountable, responsible and equitable AI use across health professions education, research and care.	Translate responsible-AI principles into role-specific practice; maintain accountability, transparency, integrity, literacy and equity.	Multiple actors	No	Partial/indirect	Yes	Moderate	5	5	5	5	Abstract and final 10-principle framework.	Governance framework	Anchor source; major general-HPE framework our anatomy framework must extend rather than duplicate.
5	IFAA 2023 Images	International Federation of Associations of Anatomists (IFAA), Federative International Committee for Ethics and Medical Humanities. Recommendations for Good Practice Around Human Tissue Image Acquisition and Use in Anatomy Education and Research. 2023.	2023	International	https://ifaa.net/committees/ethics-and-medical-humanities-ficem/recommendations-for-good-practice-around-human-tissue-image-acquisition-and-use-in-anatomy-education-and-research/	Professional guideline	Guidance/policy	No	Anatomy ethics/human tissue imaging	Yes	Professional/normative guidance	Not applicable (non-AI source)	Resource generation	Human tissue image	2. Input & donor-derived material governance	Digital acquisition, storage, distribution and reuse of images derived from human tissues can detach images from consented educational purposes.	Consent, dignity, privacy, secure storage, appropriate distribution and institutional oversight.	Loss of donor dignity, privacy breaches, misuse, uncontrolled redistribution and erosion of public trust.	Body donor/donor family	Recommends purpose-appropriate consent, protection against identification, restricted distribution, secure institutional storage, avoidance of non-institutional commercial servers as best practice, and anatomical oversight of image governance.	Verify consent; redact identifiers; restrict access; store securely on institutional systems; establish transparent oversight for acquisition, storage, use and destruction.	Ethics/body donation committee	Yes	No	Partial/indirect	Restricted/Avoid	5	5	5	5	Guideline items 2-5 and 11-14.	Donor-derived materials	Anchor normative source; highly relevant to uploading donor-derived material to third-party GenAI platforms.
6	Jones, 2026 IFAA	Jones DG, Champney TH, Hildebrandt S, et al. IFAA Recommendations for good practice for the donation and anatomical study of human remains (revised 2026). Anatomical Sciences Education. 2026 Jul 1. Online ahead of print. doi:10.1002/ase.70289.	2026	International	https://doi.org/10.1002/ase.70289	Professional guideline	Online first	Yes	Anatomy ethics/body donation governance	Yes	Professional/normative guidance	Not applicable (non-AI source)	Institutional governance	Cadaveric/donor image	Cross-cutting/multiple	Contemporary body-donation practice requires updated institutional standards reflecting evolving sociocultural and technological expectations.	Ethical acquisition, stewardship, governance and sustainable use of human remains.	Ethically inconsistent handling of donated human remains and loss of public trust.	Body donor/donor family	Revised IFAA recommendations provide an updated international framework for institutions using human remains in anatomy education and research.	Align institutional body-donation and human-remains policies with contemporary ethical recommendations; maintain formal governance and oversight.	Institution/medical school	Yes	No	Partial/indirect	High	5	5	4	4.7	PubMed abstract; revised 2026 international recommendations.	Donor-derived materials	Anchor normative source; establishes current international ethical baseline for donor-derived materials.
7	Cornwall, 2016	Cornwall J, Callahan D, Wee R. Ethical issues surrounding the use of images from donated cadavers in the anatomical sciences. Clinical Anatomy. 2016;29(1):30-36. doi:10.1002/ca.22644.	2016	New Zealand/Portugal	https://doi.org/10.1002/ca.22644	Ethics analysis	Published	Yes	Anatomy ethics/cadaveric images	Yes	Conceptual/ethical scholarship	Not applicable (non-AI source)	Resource generation	Cadaveric/donor image	2. Input & donor-derived material governance	Cadaver images can be copied, distributed and commercialized in ways that may not match donor expectations.	Consent, social trust, appropriate educational use and institutional stewardship.	Unconsented use or misuse of cadaveric images may harm donor interests and body-donation programs.	Body donor/donor family	Argues that cadaver-image governance is central to maintaining donor/public trust and that informed consent is necessary for images arising from donated cadavers.	Use explicit informed consent and responsible institutional governance for acquisition and use of cadaveric images.	Ethics/body donation committee	Yes	No	No	High	5	4	5	4.7	Abstract; discussion of donor expectations, consent, misuse and public trust.	Donor-derived materials	Foundational ethics source for donor-image governance.
8	Iwanaga, 2025	Iwanaga J, Kim HJ, Akita K, et al. Ethical Use of Cadaveric Images in Anatomical Textbooks, Atlases, and Journals: A Consensus Response From Authors and Editors. Clinical Anatomy. 2025;38(2):222-225. doi:10.1002/ca.24258.	2025	International	https://doi.org/10.1002/ca.24258	Editorial/perspective	Published	Yes	Anatomy ethics/publishing	Yes	Conceptual/ethical scholarship	Not applicable (non-AI source)	Resource generation	Cadaveric/donor image	6. Provenance, IP & disclosure	Whether professional guidance requiring specific consent for commercial cadaveric-image use is too restrictive.	Balance between donor protection, educational access, historical resources and professional discretion.	Overly restrictive image rules may limit legitimate anatomy education; overly permissive use may still create ethical concerns.	Multiple stakeholders	Senior anatomy authors/editors challenge aspects of IFAA image guidance and argue that anonymized cadaveric images used in good-faith educational resources may not require additional consent beyond traditional donation consent.	Distinguish professional guidance from binding law; preserve ethical consent processes while avoiding claims that contested recommendations are universally settled.	Multiple actors	Yes	No	No	High	5	4	5	4.7	Abstract and discussion responding to 2023 IFAA image guidance.	Donor-derived materials	Anchor counterpoint; essential for presenting donor-image ethics as contested rather than settled.
9	Eldesoqui, 2025	Eldesoqui M, Albadawi EA, AlQumaizi KI, Radwan MNM, Ebrahim HA, Elsaid MAE. Assessing the Anatomical Accuracy of AI-Generated Medical Illustrations: A Comparative Study of Text-to-Image Generator Tools in Anatomy Education. Clinical Anatomy. 2025;38(6):712-717. doi:10.1002/ca.70002.	2025	Saudi Arabia/Egypt	https://doi.org/10.1002/ca.70002	Original empirical research	Published	Yes	Anatomy education	Yes	Direct anatomy evidence	Text-to-image	Anatomical image generation	AI-generated image	3. Anatomical fidelity & source verification	Text-to-image generators can produce visually plausible but structurally incorrect anatomical illustrations.	Accuracy, educational validity and limits of substituting AI for expert medical illustration.	Learners may encode incorrect anatomy from inaccurate visual exemplars.	Learner	Four text-to-image tools showed variable anatomical performance; errors included sulcal/gyral discrepancies, incorrect rib counts and inaccurate hand skeletons, with significant limitations especially for bony structures.	Require expert anatomical review and continuous assessment before AI-generated illustrations are used for medical education.	Educator/faculty	No	No	Yes	High	5	4	5	4.7	PubMed abstract; specific heart, brain, thorax and hand findings.	Anatomical fidelity	Anchor direct empirical evidence for fidelity/verification domain.
10	Alon, 2026	Alon L, Shoval DH, Levkovich I. Bias, representation, and clinical fidelity in AI-generated images for medical education: a systematic literature review. npj Digital Medicine. 2026;9:469. doi:10.1038/s41746-026-02608-3.	2026	Israel	https://doi.org/10.1038/s41746-026-02608-3	Systematic review	Published	Yes	Medical education/AI-generated images	No	Transferable HPE evidence	Text-to-image	Anatomical image generation	AI-generated image	4. Variation, bias & representation	AI-generated medical images may combine demographic bias with clinically/anatomically implausible content.	Fairness, representational coverage, fidelity, learner trust and safe educational use.	Biased prototypes and plausible but incorrect images may anchor incorrect mental models.	Learner	Across 36 empirical studies, representational bias was common and clinical-fidelity limitations included anatomical hallucinations; the review argues AI-generated images require active governance and expert curation.	Use use-sensitive validation, expert curation, representation audits and visual AI literacy before educational deployment.	Institution/medical school	No	Partial/indirect	Yes	High	5	5	5	5	Abstract and practical-guidance sections; 36-study synthesis.	Bias/representation	Anchor high-level evidence for image fidelity, bias and proportional governance.
11	Gupta, 2026	Gupta K, Ferri Latinovich M, Ferri Latinovich M, Singh K, Patlas MN, Jajodia A. Generative Artificial Intelligence for Medical Image Creation in Health Professions Education: a Scoping Review. Journal of Medical Systems. 2026;50(1):19. doi:10.1007/s10916-026-02350-z.	2026	International	https://doi.org/10.1007/s10916-026-02350-z	Scoping review	Published	Yes	Health professions education/medical imaging	No	Transferable HPE evidence	Text-to-image	Resource generation	AI-generated image	3. Anatomical fidelity & source verification	Generative medical-image tools are entering education without consistent validation for fidelity, safety or equity.	Educational reliability, safety, equity and validation.	Unvalidated synthetic images may be used as teaching resources despite uncertain accuracy and educational impact.	Learner	Scoping review identified 19 studies; applications included anatomy, but study designs and validation approaches were heterogeneous and evidence for educational fidelity/safety remained limited.	Treat synthetic images as provisional educational resources requiring domain-specific validation and oversight.	Educator/faculty	No	Partial/indirect	Yes	High	4	5	4	4.3	PubMed abstract; 19-study scoping review.	Anatomical fidelity	Anchor mapping review for broader image-generation evidence and gaps.
12	Agarwal, 2026	Agarwal P, Agarwal R, Iezhitsa I. AI for Assessment in Medical Education in Post LLM Era: A Scoping Review. Medical Science Educator. 2026;36(2):1027-1039. doi:10.1007/s40670-026-02653-3.	2026	Malaysia	https://doi.org/10.1007/s40670-026-02653-3	Scoping review	Published	Yes	Medical education assessment	No	Transferable HPE evidence	Generative assessment tool	Question generation	Assessment content	5. Assessment validity & integrity	AI-generated assessment content can be efficient but may contain shallow reasoning, weak distractors, factual inaccuracies and reduced discriminatory power.	Assessment validity, reliability, fairness and responsible human-in-the-loop use.	Poor-quality or insufficiently reviewed AI-generated assessment items may weaken validity and misclassify learner performance.	Learner	Review of 25 studies supports AI as assistive rather than substitutive in assessment and calls for standardized human-in-the-loop workflows and post-exam psychometric assurance.	Require expert review; maintain human-in-the-loop assessment design; apply post-exam psychometric checks for consequential use.	Assessment committee	No	Yes	Yes	High	4	5	4	4.3	PubMed abstract; 25-study review and conclusions.	Assessment	Anchor transferable evidence for anatomy assessment governance.
13	Che Liu, 2026	Che X, Liu Y. Guiding student use of generative AI in undergraduate pharmacology: a narrative review and proposed source-checking process framework. Frontiers in Medicine. 2026;13:1910543. doi:10.3389/fmed.2026.1910543.	2026	China	https://doi.org/10.3389/fmed.2026.1910543	Narrative review	Published	Yes	Undergraduate pharmacology/health professions education	No	Transferable HPE evidence	Large language model	Learning support	Public text	3. Anatomical fidelity & source verification	Fluent AI outputs can conceal unsupported or incorrect discipline-specific claims, while final-answer assessment may hide the student's verification process.	Source integrity, transparency, visible reasoning and learner accountability.	Unchecked AI claims can be submitted or learned without demonstrating disciplinary reasoning.	Learner	Proposes a five-step source-checking process: define/record AI use, verify claims against defined sources, rebuild mechanism, test against context, and revise with feedback.	Make verification visible; define trusted source sets; document AI use; require reasoning and correction rather than accepting final output at face value.	Learner	No	Partial/indirect	Yes	Moderate	4	4	5	4.3	Frontiers abstract and framework section; five-step source-checking process.	Governance framework	Anchor discipline-specific operational precedent; useful model for anatomy verification workflow.
14	WHO 2024 LMM	World Health Organization. Ethics and governance of artificial intelligence for health: Guidance on large multi-modal models. Geneva: World Health Organization; 2024.	2024	Global	https://www.who.int/publications/i/item/9789240084759	Professional guideline	Guidance/policy	No	Health AI governance	No	Professional/normative guidance	Multimodal generative model	Institutional governance	Other/unclear	7. Human oversight & accountability	Large multimodal generative models introduce governance challenges across data inputs, generated outputs and health-related uses.	Safety, autonomy, accountability, transparency, privacy and responsible governance.	Unvalidated or poorly governed multimodal AI may cause harm when outputs are trusted beyond their demonstrated capabilities.	Multiple stakeholders	WHO provides ethics and governance guidance specifically for large multimodal models used in health-related contexts.	Use human-centered governance, appropriate oversight and safeguards proportionate to use and risk; do not assume general-purpose models are fit for sensitive health tasks.	Institution/medical school	Partial/indirect	Partial/indirect	Yes	High	4	5	4	4.3	WHO guidance, publication copyright/suggested citation: 2024; current WHO item page may display a later webpage date.	Governance framework	Anchor global normative source for multimodal GenAI governance; transferred cautiously to education. Bibliographic year corrected to 2024 based on WHO publication metadata; the live WHO item page has displayed a 2025 webpage date.
16	Abdellatif, 2022	Abdellatif H, Al Mushaiqri M, Albalushi H, Al-Zaabi AA, Roychoudhury S, Das S. Teaching, Learning and Assessing Anatomy with Artificial Intelligence: The Road to a Better Future. Int J Environ Res Public Health. 2022;19(21):14209. doi:10.3390/ijerph192114209.	2022	Oman	https://doi.org/10.3390/ijerph192114209	Narrative review	Published	Yes	Anatomy education	Yes	Conceptual/ethical scholarship	Conventional AI (foundational only)	Other	No sensitive input	Cross-cutting/multiple	Broad AI adoption in anatomy teaching, learning and assessment raises practical questions about appropriate educational integration and preservation of core anatomy learning.	Appropriate educational use, preservation of educator roles, and balanced adoption of technology.	Over-reliance on technology or inappropriate substitution for established anatomy teaching methods.	Multiple stakeholders	Early anatomy-specific review mapping AI applications across teaching, learning and assessment and arguing for a balanced future-facing approach rather than wholesale replacement of established methods.	Use AI as an adjunct within anatomy curricula and retain educator judgment when integrating technology.	Individual educator	Partial/indirect	Yes	Partial/indirect	Moderate	4	4	3	3.7	Review overview and conclusions.	Introduction/background	S1 core-search addition; foundational pre-GenAI anatomy review.
17	Joseph, 2025	Joseph TS, Gowrie S, Montalbano MJ, Bandelow S, Clunes M, Dumont AS, Iwanaga J, Tubbs RS, Loukas M. The Roles of Artificial Intelligence in Teaching Anatomy: A Systematic Review. Clinical Anatomy. 2025;38(5):552-567. doi:10.1002/ca.24272.	2025	Grenada/international collaboration	https://doi.org/10.1002/ca.24272	Systematic review	Published	Yes	Gross anatomy education	Yes	Direct anatomy evidence	General AI/mixed modalities	Learning support	No sensitive input	Cross-cutting/multiple	AI applications in anatomy span simulation, adaptive feedback and personalized learning, but implementation can create over-reliance, bias and diminished human interaction.	Balanced integration, educational oversight, preservation of human interaction and attention to bias.	Over-reliance, biased outputs or reduced human interaction may undermine educational quality.	Multiple stakeholders	Systematic review included 61 studies and concluded that AI may enhance engagement and personalization, while emphasizing ethical and practical implications and the need for balanced implementation with robust oversight.	Combine AI with traditional methods and maintain robust educational oversight rather than treating AI as a replacement.	Anatomy department	Partial/indirect	Partial/indirect	Partial/indirect	Moderate	5	5	5	5	Abstract; systematic-review methods/results and conclusion.	Competitor/novelty analysis	S1 core-search addition; major broad anatomy-AI review and important competitor.
18	Chytas, 2025	Chytas D, Noussios G, Paraskevas G, Vasiliadis AV, Giovanidis G, Troupis T. Can ChatGPT play a significant role in anatomy education? A scoping review. Morphologie. 2025;109(365):100949. doi:10.1016/j.morpho.2025.100949.	2025		https://doi.org/10.1016/j.morpho.2025.100949	Scoping review	Published	Yes	Anatomy education	Yes	Direct anatomy evidence	Large language model	Learning support	Public text	3. Anatomical fidelity & source verification	ChatGPT-generated anatomy information may be incomplete, inaccurate or scientifically weak despite educational potential.	Reliability, accuracy and safe use of generated educational information.	Learners may rely on weak or scientifically unsound anatomy outputs.	Learner	Nine studies were included; the review reported weak ChatGPT performance for anatomical questions and failures to generate detailed answers, acceptable images and scientifically sound texts, while noting some educational utility.	Treat generated content as provisional and subject it to expert/source verification before educational use.	Educator/faculty	No	Partial/indirect	Yes	High	5	4	4	4.4	Abstract and conclusion; nine-study scoping review.	Anatomical fidelity	S1 addition; also belongs to S3 fidelity stream.
19	Erbek, 2026	Erbek E, Çalışkan Pala S, Bolatlı G, Çavuş F. Artificial intelligence in anatomy education: a systematic review of ChatGPT's effectiveness as a learning tool. Proc (Bayl Univ Med Cent). 2026;39(2):315-323. doi:10.1080/08998280.2026.2612683.	2026	Türkiye	https://doi.org/10.1080/08998280.2026.2612683	Systematic review	Published	Yes	Anatomy education	Yes	Direct anatomy evidence	Large language model	Learning support	Public text	3. Anatomical fidelity & source verification	Performance and reliability vary substantially by model version and anatomical topic, limiting uncritical educational use.	Reliability, reproducibility and appropriate evidence-based use of LLM-generated anatomy content.	Variable model accuracy may expose learners to incorrect or inconsistent anatomical information.	Learner	Systematic review retained 23 studies and found wide accuracy ranges across ChatGPT model versions; it concluded that ChatGPT may be complementary but requires careful evaluation and further evidence.	Require version-aware evaluation and expert verification; avoid treating LLM output as an authoritative anatomy source.	Educator/faculty	No	Partial/indirect	Yes	High	5	5	5	5	Abstract; results and conclusion.	Competitor/novelty analysis	S1 addition; direct 2026 systematic review competitor, also S3.
20	Collins, 2024 Anatomy GPT	Collins BR, Black EW, Rarey KE. Introducing AnatomyGPT: A customized artificial intelligence application for anatomical sciences education. Clinical Anatomy. 2024;37(6):661-669. doi:10.1002/ca.24178.	2024	United States	https://doi.org/10.1002/ca.24178	Original empirical research	Published	Yes	Anatomical sciences education	Yes	Direct anatomy evidence	Large language model	Learning support	Public text	1. Use-case & risk classification	Custom GPTs can be configured as anatomy learning tools, creating a need to define acceptable educational uses, source grounding and faculty responsibility.	Educational reliability, appropriate configuration and faculty oversight.	A customized tutor may be perceived as authoritative even when its limitations and source boundaries are not understood.	Learner	The study describes and explores AnatomyGPT as a customized GPT for anatomical sciences education and concludes that custom GPTs may have educational potential while requiring further development and characterization.	Define intended use, source boundaries and faculty oversight before deploying customized anatomy GPTs.	Educator/faculty	No	No	Yes	Moderate	4	4	4	4	Abstract and article conclusion.	Introduction/background	S1 addition; practical GenAI implementation example.
21	Temizsoy Korkmaz, 2025	Temizsoy Korkmaz F, Ok F, Karip B, Keleş P. A structured evaluation of LLM-generated step-by-step instructions in cadaveric brachial plexus dissection. BMC Medical Education. 2025;25:903. doi:10.1186/s12909-025-07493-0.	2025	Türkiye	https://doi.org/10.1186/s12909-025-07493-0	Original empirical research	Published	Yes	Cadaveric anatomy education/surgical anatomy	Yes	Direct anatomy evidence	Large language model	Teaching explanation	Public text	3. Anatomical fidelity & source verification	LLMs used as procedural dissection guides operate in a low-error-tolerance environment where anatomical accuracy, spatial fidelity and practical usability must be evaluated.	Safety of procedural guidance, accuracy, educational quality and limits of AI use in cadaveric instruction.	Incorrect procedural or spatial guidance could waste scarce donor material or teach unsafe/incorrect dissection approaches.	Multiple stakeholders	Four LLMs were evaluated on 28 brachial-plexus dissection questions by experienced anatomists for scientific quality, educational value and readability, directly testing LLM guidance in cadaveric anatomy.	Require expert anatomical validation and restrict procedural LLM guidance to complementary, supervised use in cadaveric education.	Educator/faculty	Yes	No	Yes	High	5	5	5	5	Abstract and methods; 28-item blinded expert evaluation.	Anatomical fidelity	S1 addition; highly valuable direct evidence, also central to S3.
22	Topcu, 2026 Cadaver AI	Topcu I, Karip B, Demir P, et al. Exploring the moral status of cadavers through artificial intelligence: a thematic analysis of AI-based perspectives in anatomy education. Philosophy, Ethics, and Humanities in Medicine. 2026;21:12. doi:10.1186/s13010-026-00222-5.	2026	Türkiye	https://doi.org/10.1186/s13010-026-00222-5	Original empirical research	Published	Yes	Anatomy education/medical ethics	Yes	Direct anatomy evidence	Large language model	Learning support	Public text	2. Input & donor-derived material governance	LLM-mediated discussion of cadavers raises questions about whether AI reproduces or distorts ethical norms concerning donor dignity, responsibility and professional conduct.	Donor dignity, professional identity, privacy, respect and appropriate ethical framing.	Anthropomorphic or uncritical reliance on AI-generated ethical reasoning may misrepresent contested norms or create false authority.	Body donor/donor family	Thematic analysis of four LLMs found recurring themes of donor terminology, educational importance and moral/ethical responsibilities; the study suggests possible educational use while acknowledging the need for ethical alignment.	Use LLM ethical outputs only as prompts for supervised reflection, not as authoritative policy or ethics guidance.	Educator/faculty	Yes	No	Partial/indirect	Moderate	4	4	4	4	Abstract; methods/results on four LLMs and cadaver moral status.	Donor-derived materials	S1 addition; anatomy-specific ethics evidence. Its methodological premise should be interpreted cautiously because LLM outputs do not themselves establish ethical norms.
23	Morgan, 2026, Assessment Literacy	Morgan A, Crabtree M, Sachsenmeier C, Troy A. Harnessing Generative Artificial Intelligence to Teach Assessment Literacy in Anatomy. Medical Science Educator. 2026;36:3-4. doi:10.1007/s40670-026-02659-x.	2026	United States	https://doi.org/10.1007/s40670-026-02659-x	Other	Published	Yes	Anatomy education/assessment literacy	Yes	Direct anatomy evidence	Generative assessment tool	Question generation	Assessment content	5. Assessment validity & integrity	Using GenAI for anatomy item writing requires learners to recognize that generated questions must be appraised and revised rather than accepted at face value.	Assessment quality, learner AI literacy and responsible human review.	Poorly appraised generated questions may reinforce weak item-writing practices or inaccurate anatomy.	Learner	An anatomy bootcamp and MBS item-writing activity explicitly taught prompt engineering plus critical appraisal and revision of AI-generated questions.	Pair GenAI item generation with explicit appraisal, revision and faculty oversight.	Educator/faculty	No	Yes	Yes	Moderate	4	4	5	4.3	Abstract and innovation description.	Assessment	S1 addition; also central to S5 assessment stream.
24	Almamari, 2026	Almamari A, Al-Farsi M, Almamari A-R, Abdellatif H. The Perceived Effectiveness of AI-Powered Tools in Undergraduate Anatomy Education: A Cross-Sectional Multifaceted Evaluation. Clinical Anatomy. 2026. Online ahead of print. doi:10.1002/ca.70188.	2026	Oman	https://doi.org/10.1002/ca.70188	Original empirical research	Online first	Yes	Undergraduate anatomy education	Yes	Direct anatomy evidence	General generative AI	Learning support	Assessment content	Cross-cutting/multiple	AI-generated anatomy content and MCQs may be useful yet learner confidence in accuracy, AI competence and ethical use remains limited.	Accuracy, human review, learner competence, ethical use and long-term educational effects.	Uncritical use may create false confidence, weak retention or acceptance of inaccurate material.	Learner	A 2025 multi-phase study combined expert content-validity review, student evaluation of AI-generated MCQs and a student survey; findings supported utility but emphasized human-in-the-loop review, structured guidance and expert validation.	Use structured guidance, expert validation and human-in-the-loop workflows for learner-facing anatomy AI tools.	Educator/faculty	No	Yes	Yes	Moderate	5	4	5	4.7	PubMed abstract; three-part design and human-in-the-loop conclusion.	Governance framework	S1 addition; newest direct anatomy evidence located in this batch (online July 15, 2026).
25	Bolgova Mavrych, 2025	Bolgova O, Mavrych V. Evolution of AI in anatomy education study based on comparison of current large language models against historical ChatGPT performance. Scientific Reports. 2025;15:37545.	2025	Saudi Arabia	https://www.nature.com/articles/s41598-025-22437-w	Original empirical research	Published	Yes	Anatomy education	Yes	Direct anatomy evidence	Large language model	Learning support	Assessment content	3. Anatomical fidelity & source verification	Rapid model evolution makes fixed claims about LLM anatomy performance unstable and reinforces the need for model/version-aware validation.	Reliability over time, reproducibility and avoidance of obsolete model-specific governance.	Policies based on one model/version may become outdated and create misplaced confidence.	Multiple stakeholders	The study compares contemporary LLM performance with historical ChatGPT results on anatomy questions, illustrating substantial model evolution and the temporal instability of benchmark findings.	Frame governance around use-case risk and verification requirements rather than model-specific approval; document model/version when used.	Educator/faculty	No	Yes	Yes	Moderate	4	4	5	4.3	Scientific Reports article; comparison with historical ChatGPT performance.	Limitations	S1 addition; also useful for S3 and for arguing against model-specific governance.
26	Hennessy, 2020	Hennessy CM, Royer DF, Meyer AJ, Smith CF. Social Media Guidelines for Anatomists. Anatomical Sciences Education. 2020;13(4):527-539. doi:10.1002/ase.1948.	2020	United Kingdom/United States/Australia	https://doi.org/10.1002/ase.1948	Professional guideline	Published	Yes	Anatomy education/social media	Yes	Professional/normative guidance	Not applicable (non-AI source)	Resource generation	Cadaveric/donor image	6. Provenance, IP & disclosure	Public sharing of cadaveric material can detach images from educational context and create uncertainty about consent, professionalism and donor provenance.	Informed consent, donor dignity, professionalism, public trust and contextual integrity.	Unauthorized or decontextualized sharing may disrespect donors, erode public trust and normalize weak consent practices.	Body donor/donor family	The paper documents controversy around cadaveric material on social media and proposes explicit professional guidance, including transparency about informed consent when such material is shared.	Require documented consent/provenance, preserve educational context, use professional accounts/policies, and avoid public dissemination when authorization is uncertain.	Individual educator	Yes	No	Partial/indirect	High	5	4	5	4.7	Abstract and guideline recommendations on cadaveric material, consent and social media.	Donor-derived materials	S2 addition; direct anatomy guidance for digital dissemination. Foundational for GenAI because public-platform upload is another form of external digital dissemination.
27	HTA 2023 Filming	Human Tissue Authority. Filming or photographing of bodies or body parts that have been donated. Last updated 11 October 2023. United Kingdom.	2023	United Kingdom	https://www.hta.gov.uk/anatomy/filming	Professional guideline	Guidance/policy	No	Anatomy regulation/body donation	Yes	Professional/normative guidance	Not applicable (non-AI source)	Resource generation	Cadaveric/donor image	2. Input & donor-derived material governance	Photography and filming of donated bodies fall outside direct statutory regulation in this context, but establishments are expected to maintain dignity and prevent inappropriate image use.	Donor dignity, prior consent, appropriate purpose and institutional responsibility.	Images may be captured or disseminated beyond the donor's intended purpose, causing dignity and trust harms.	Body donor/donor family	HTA guidance states that institutions should prevent inappropriate use of donated-body images and that prior consent for filming, photography and subsequent uses should ideally be obtained from the donor.	Require purpose-specific consent checks and institutional authorization before capturing, transforming, uploading or disseminating donor-derived images.	Institution/medical school	Yes	No	Partial/indirect	High	5	5	5	5	Official HTA guidance; sections on dignity, inappropriate use and prior consent.	Donor-derived materials	S2 addition; jurisdiction-specific UK regulatory guidance. It should inform, not be presented as universal law.
28	HTA 2024 Code A	Human Tissue Authority. Code A: Guiding principles and the fundamental principle of consent — Section three: Consent requirements. Updated 2024. United Kingdom.	2024	United Kingdom	https://www.hta.gov.uk/guidance-professionals/codes-practice-standards-and-legislation/codes-practice/code-guiding-principles-and-fundamental-principle-consent/code-section-three-consent-requirements	Policy/framework document	Guidance/policy	No	Human tissue regulation/anatomy	Yes	Professional/normative guidance	Not applicable (non-AI source)	Resource generation	Human tissue image	2. Input & donor-derived material governance	Electronic images may sit outside specific tissue legislation even though institutional systems remain necessary to preserve dignity and prevent inappropriate use.	Consent, dignity, governance beyond minimum legal compliance and appropriate use of images.	A legal-regulatory gap could be misread as ethical permission for unrestricted digital use.	Institution	The HTA distinguishes legal scope from institutional responsibility: images can fall outside the Human Tissue Act while establishments are still expected to maintain dignity and prevent inappropriate image use.	Treat legal permissibility as a floor, not the complete governance standard; require institutional policies for digital and AI-related uses of donor-derived material.	Institution/medical school	Yes	No	Partial/indirect	High	5	5	4	4.7	Code A, section 'Use of images' and consent requirements for deceased tissue.	Institutional governance	S2 addition; useful for our manuscript's distinction between law, professional guidance, ethics and institutional policy.
29	Passalacqua, 2025	Passalacqua NV, Bartelink E, McQuade WEP, Steadman D, Boyd D, Spradley K, Sauerwein K, Ho R. The Development of Standards for the Ethical Use of Human Skeletal Remains for Education, Research, and Training in Forensic Anthropology. American Journal of Biological Anthropology. 2025;186(3):e70022. doi:10.1002/ajpa.70022.	2025	United States	https://doi.org/10.1002/ajpa.70022	Consensus statement	Published	Yes	Forensic anthropology/human remains education	Partial/indirect	Professional/normative guidance	Not applicable (non-AI source)	Other	Human tissue image	2. Input & donor-derived material governance	Ethical permission for human-remains use depends on origin and consent, and the same permissions matter for associated images, measurements and data.	Informed consent, provenance, community interests, respectful treatment and downstream data use.	Digital or associated data can be separated from the governance conditions attached to the physical remains.	Multiple stakeholders	The proposed standard grounds ethical use in informed consent, deathcare and service to communities and explicitly extends governance to information associated with remains, including images, measurements and data.	Carry provenance and consent conditions forward to digital derivatives; document origin and permitted uses before reuse or external processing.	Institution/medical school	Yes	No	Partial/indirect	High	4	5	4	4.3	Abstract and proposed-standard discussion of origin-based consent plus associated images/data.	Donor-derived materials	S2 addition; adjacent forensic anthropology rather than medical anatomy, so use as transferable human-remains governance evidence.
30	Keet and Kramer, 2022	Keet K, Kramer B. Advances in Digital Technology in Teaching Human Anatomy: Ethical Predicaments. Advances in Experimental Medicine and Biology. 2022;1388:173-191. doi:10.1007/978-3-031-10889-1_8.	2022	South Africa	https://doi.org/10.1007/978-3-031-10889-1_8	Ethics analysis	Published	Unclear	Anatomy education/digital technology	Yes	Conceptual/ethical scholarship	Not applicable (non-AI source)	Resource generation	Cadaveric/donor image	Cross-cutting/multiple	Digital photography, video, 3D printing and online distribution create unresolved questions about provenance, permission, privacy, ownership, commercialization and oversight.	Consent, privacy of the deceased, ownership, commercialization, context and institutional accountability.	Digitized donor-derived material can be copied, transformed or redistributed beyond original educational intent.	Body donor/donor family	The chapter argues that digital anatomy makes ethical sourcing and use increasingly urgent and explicitly asks who should oversee digital and 3D-printed models derived from human bodies.	Establish institutional oversight of digital derivatives, verify provenance/permission, and preserve restrictions when material is copied, transformed or redistributed.	Anatomy department	Yes	No	Partial/indirect	High	5	4	5	4.7	PubMed abstract; digital images, 3D printing, privacy, ownership, commercialization and oversight.	Donor-derived materials	S2 addition; direct anatomy ethics source that predates current GenAI but maps closely onto input, transformation and dissemination governance.
31	Lottering, 2022	Lottering T, Billings B, Brits D, Hutchinson E, Kramer B. The ethical use of digital technology in teaching anatomy: A southern African perspective. Annals of Anatomy. 2022;244:151990. doi:10.1016/j.aanat.2022.151990.	2022	Southern Africa	https://doi.org/10.1016/j.aanat.2022.151990	Original empirical research	Published	Yes	Anatomy education/digital resources	Yes	Direct anatomy evidence	Not applicable (non-AI source)	Resource generation	Human tissue image	6. Provenance, IP & disclosure	Anatomy educators may use externally sourced or departmental digital human-material resources without reliably knowing provenance or consent conditions.	Provenance, informed consent, use of unclaimed bodies, educator responsibility and transparency.	Unknown provenance can normalize ethically questionable material and make educators unable to assure learners or donors that use is authorized.	Multiple stakeholders	Survey findings show provenance-awareness gaps among anatomy educators using digital resources and reported ethical conflict around images derived from unclaimed bodies.	Require provenance metadata and consent status for human-derived digital resources before they enter teaching collections or AI workflows.	Anatomy department	Yes	No	Partial/indirect	High	5	4	5	4.7	Abstract/highlights; provenance awareness and conflict regarding unclaimed-body images.	Donor-derived materials	S2 addition; direct empirical evidence that provenance controls are not merely theoretical.
32	Cornwall, 2025 Illustrations	Cornwall J, White R, Pennefather P, Hildebrandt S, Gregory J, Smith HF, Organ J, Krebs C. Legal and ethical considerations around the use of existing illustrations to generate new illustrations in the anatomical sciences. Anatomical Sciences Education. 2025;18(3):289-300. doi:10.1002/ase.70002.	2025	New Zealand/Canada/United States	https://doi.org/10.1002/ase.70002	Ethics analysis	Published	Yes	Anatomical sciences/illustration/GenAI	Yes	Conceptual/ethical scholarship	Text-to-image	Anatomical image generation	Educational image	6. Provenance, IP & disclosure	Generating new anatomical illustrations from existing works creates copyright, plagiarism and provenance problems, which are amplified by generative AI.	Copyright, fair use/fair dealing, plagiarism, attribution, originality and visual provenance.	AI-generated or derivative illustrations may reproduce protected expression, obscure source lineage or misrepresent originality.	Multiple stakeholders	The article provides anatomy-specific legal/ethical guidance for derivative illustrations and highlights the absence of a simple bright-line test for originality, with explicit relevance to generative AI.	Document source provenance; obtain permissions where required; review copyright/plagiarism risk; disclose derivation and avoid presenting uncertain derivative work as original.	Educator/faculty	Partial/indirect	No	Yes	Moderate	5	5	5	5	Abstract and decision guidance on copyright, plagiarism, originality and generative AI.	Governance framework	S2 addition; major direct source for Domain 6 and one of the strongest anatomy-specific GenAI governance papers adjacent to our proposed framework.
33	Ho, 2026 YouTube	Ho A, Hulme A, Prvan T, Štrkalj G. Evidence for an ethics-practice gap in digital anatomy education: Provenance and consent disclosure in English-language YouTube videos. Annals of Anatomy. 2026;266:152856. doi:10.1016/j.aanat.2026.152856.	2026	Australia	https://doi.org/10.1016/j.aanat.2026.152856	Original empirical research	Published	Yes	Digital anatomy education/YouTube	Yes	Direct anatomy evidence	Not applicable (non-AI source)	Resource generation	Human tissue image	6. Provenance, IP & disclosure	Publicly accessible digital anatomy can omit basic provenance and consent information even when professional recommendations call for such disclosure.	Transparency, donor consent, provenance, platform accountability and professional self-regulation.	Human-tissue content may circulate globally without viewers knowing source, consent status or authorization, undermining donor dignity and accountability.	Body donor/donor family	Content analysis of 239 YouTube videos found provenance disclosed in 21.3% and multimedia-use consent documented in 4.2%, demonstrating a measurable ethics-practice gap in digital anatomy.	Require standardized provenance and consent metadata for donor-derived digital content; assign creator/institution responsibility and restrict public release when authorization is unclear.	Multiple actors	Yes	No	Partial/indirect	High	5	5	5	5	Abstract/results: 239 videos; provenance 21.3%; multimedia-use consent 4.2%.	Donor-derived materials	S2 addition; strongest direct empirical evidence located for a real-world digital anatomy governance failure.
34	Rossetti, 2026 Body Data	Rossetti N, Licata M, Fusco R, Vanni A, Picozzi M. The Body as Data: from human remains to digital files. A review of current literature. Digital Applications in Archaeology and Cultural Heritage. 2026;41:e00543. doi:10.1016/j.daach.2026.e00543.	2026	Italy	https://doi.org/10.1016/j.daach.2026.e00543	Systematic review	Published	Yes	Digital human remains/anthropology/heritage	Partial/indirect	Conceptual/ethical scholarship	Not applicable (non-AI source)	Resource generation	Human tissue image	6. Provenance, IP & disclosure	Digitization converts human remains into reproducible data whose ownership, access, reuse, display and contextual integrity can diverge from the governance of the physical remains.	Authorization, ownership, access, reuse, cultural authority, commercialization and contextual integrity.	Digital surrogates can be copied or repurposed at scale, detaching them from consent, context and stakeholder control.	Multiple stakeholders	The 2026 review identifies recurring governance tensions around ownership, sharing, reproduction and display and proposes attention to authorization, accessibility and contextual integrity for digital human-remains data.	Treat digital derivatives as governed extensions of human remains rather than ordinary files; preserve authorization, access controls, metadata and contextual integrity across reuse.	Institution/medical school	Yes	No	Partial/indirect	High	4	5	5	4.6	Abstract/conclusions; ownership, sharing, reproduction, display; authorization-accessibility-contextual integrity.	Governance framework	S2 addition; adjacent anthropology, explicitly excludes medical education, so use as transferable conceptual evidence rather than direct anatomy-education evidence.
35	Kramer Moxham, 2026	Kramer B, Moxham B. Beyond the grave: Do the dead have rights? Anatomical Sciences Education. 2026. doi:10.1002/ase.70259.	2026	South Africa/United Kingdom	https://doi.org/10.1002/ase.70259	Editorial/perspective	Online first	Yes	Anatomical sciences ethics	Yes	Conceptual/ethical scholarship	Not applicable (non-AI source)	Resource generation	Cadaveric/donor image	2. Input & donor-derived material governance	Legal rights after death vary, but anatomists retain moral and professional duties regarding dignity, consent, privacy, images, digital derivatives and exploitation.	Posthumous dignity, privacy, autonomy, donor wishes, exploitation and professional custodianship.	Treating death as removal of all ethical constraints could justify unconsented images, digital derivatives or commercial reuse that conflict with donor wishes and public trust.	Body donor/donor family	The viewpoint argues that anatomists should protect posthumous dignity and explicitly includes digitized imagery, 3D models and unconsented images among practices requiring ethical scrutiny.	Separate legal-rights analysis from professional moral duties; make donor wishes, dignity, privacy and non-exploitation explicit governance constraints.	Anatomy department	Yes	No	Partial/indirect	High	5	4	5	4.7	Abstract/introduction/recommendations; digitized imagery, privacy, unconsented images and 3D models.	Discussion	S2 addition; very recent anatomy ethics source. Present as a viewpoint and acknowledge ongoing disagreement over posthumous rights.
36	Iwanaga, 2022 Statement	Iwanaga J, Singh V, Takeda S, et al. Standardized statement for the ethical use of human cadaveric tissues in anatomy research papers: Recommendations from Anatomical Journal Editors-in-Chief. Clinical Anatomy. 2022;35(4):526-528. doi:10.1002/ca.23849.	2022	International	https://doi.org/10.1002/ca.23849	Consensus statement	Published	Yes	Anatomical research/donor acknowledgement	Yes	Professional/normative guidance	Not applicable (non-AI source)	Other	Cadaveric/donor image	6. Provenance, IP & disclosure	Ethical use of cadaveric donors is inconsistently acknowledged in anatomical scholarship, creating a need for standardized disclosure.	Transparency, donor acknowledgement, consistency of ethical reporting and respect for donors.	Opaque or inconsistent reporting can obscure how donor material was sourced and ethically governed.	Body donor/donor family	Editors-in-chief from multiple anatomical journals developed standardized language to improve disclosure and acknowledgement of ethical cadaver use.	Use standardized provenance/ethics disclosure for donor-derived resources and extend analogous disclosure to digital or AI-mediated derivatives where appropriate.	Publisher/journal	Yes	No	Partial/indirect	Moderate	4	5	4	4.3	Abstract; rationale for standardized ethical-use statements in anatomy publications.	Donor-derived materials	S2 addition; reporting-focused rather than AI-focused, but useful precedent for standardizing provenance and ethical disclosure.
37	Hyeamang, 2025	Hyeamang LJ, Sekhar TC, Rush E, Beresheim AC, Cheverko CM, Brooks WS, et al. AI's ability to interpret unlabeled anatomy images and supplement educational research as an AI rater. Anatomical Sciences Education. 2025;18(10):1102-1113. doi:10.1002/ase.70074.	2025	United States/Australia	https://doi.org/10.1002/ase.70074	Original empirical research	Published	Yes	Anatomy education/image interpretation	Yes	Direct anatomy evidence	Multimodal generative model	Learning support	Educational image	3. Anatomical fidelity & source verification	Commercial multimodal GenAI may misidentify unlabeled anatomical structures and may be unreliable as an autonomous rater.	Accuracy and reliability of learner-facing anatomical interpretation and automated evaluation.	Incorrect identification or explanation of anatomical structures; false confidence in AI-generated ratings.	Learner	Across 45 anatomy-image outputs, factual accuracy was 68.0% for ChatGPT-4o and 61.5% for Claude 3.5 Sonnet; an AI rater showed only moderate-to-substantial agreement with human anatomy experts.	Require human anatomy-expert verification before learner-facing use of multimodal outputs; do not use an AI rater as the sole quality-control mechanism.	Individual educator	No	Partial/indirect	Yes	High	5	5	5	5	PubMed abstract: factual accuracy 68.0% vs 61.5%; AI-rater kappa 0.545-0.755.	Anatomical fidelity	Strong direct evidence that image interpretation and AI-based rating remain imperfect.
38	Mavrych, 2025 Gross Anatomy	Mavrych V, Ganguly P, Bolgova O. Using large language models (ChatGPT, Copilot, PaLM, Bard, and Gemini) in Gross Anatomy course: Comparative analysis. Clinical Anatomy. 2025;38(2):200-210. doi:10.1002/ca.24244.	2025	Saudi Arabia	https://doi.org/10.1002/ca.24244	Original empirical research	Published	Yes	Gross anatomy education	Yes	Direct anatomy evidence	Large language model	Teaching explanation	Assessment content	3. Anatomical fidelity & source verification	Performance varies substantially across LLMs and repeated attempts on gross-anatomy questions and generated teaching content.	Reliability, model variability, and safe use of generated anatomy explanations/questions.	Learners or educators may accept inaccurate or incomplete gross-anatomy content as authoritative.	Multiple stakeholders	On 50 USMLE-style anatomy MCQs, ChatGPT-4 achieved 60.5% mean accuracy, while other tested models performed lower; generated clinical scenarios/MCQs also varied in quality.	Treat model output as draft educational material requiring expert review; do not infer reliability from fluency or brand/model name alone.	Individual educator	No	Yes	Yes	Moderate	5	5	5	5	PubMed abstract: ChatGPT-4 60.5% ±1.9%; other models 33.5%-42.0%; authors state LLMs cannot fully replace teacher/facilitator.	Anatomical fidelity	Useful direct evidence for model- and task-specific variability.
39	AlKhater, 2025	Al-Khater KMK. Comparative assessment of three AI platforms in answering USMLE Step 1 anatomy questions or identifying anatomical structures on radiographs. Clinical Anatomy. 2025;38(2):186-199. doi:10.1002/ca.24243.	2025	Saudi Arabia	https://doi.org/10.1002/ca.24243	Original empirical research	Published	Yes	Anatomy education/radiographic anatomy	Yes	Direct anatomy evidence	Multimodal generative model	Imaging/radiological anatomy	Medical image	3. Anatomical fidelity & source verification	LLM accuracy and explanatory quality differ by platform and task, including radiographic-structure identification.	Verification of anatomy answers and image-based identification before educational use.	Incorrect anatomical identification or explanation, particularly when educators generalize performance from a small benchmark.	Learner	In a small comparative study using knowledge, analysis, and radiograph prompts, performance differed materially across ChatGPT, Claude, and Gemini; the study supported selective use rather than assuming equivalent reliability.	Verify image-based and reasoning outputs against authoritative anatomy sources and educator review; avoid treating a single benchmark as proof of general reliability.	Individual educator	No	Partial/indirect	Yes	High	4	5	4	4.3	PubMed record; 23 prompts included 10 knowledge, 10 analysis, and 3 radiograph tasks.	Anatomical fidelity	Small sample, so useful mainly as evidence of platform/task variability.
40	Sivri, 2026 Radiological	Sivri I, Ozden FM, Celik H, Gokturk O, Colak T. Comparative evaluation of radiological anatomy knowledge and accuracy of ChatGPT-5, Gemini 2.5, and Grok 4 across normal and thinking modes. Anatomical Sciences Education. 2026 Jun 26. Online ahead of print. doi:10.1002/ase.70292.	2026	Türkiye	https://doi.org/10.1002/ase.70292	Original empirical research	Online first	Yes	Radiological anatomy education	Yes	Direct anatomy evidence	Multimodal generative model	Imaging/radiological anatomy	Medical image	3. Anatomical fidelity & source verification	Radiological-anatomy identification accuracy varies markedly by model and reasoning mode; response stability also varies.	Model variability, temporal consistency, and reliability in image-dependent anatomy.	Misidentification of structures on radiographs and inconsistent outputs across modes/time.	Learner	Across 30 radiographs, overall accuracy ranged from 47.4% to 85.7%; model rankings and performance changed by mode, and temporal stability was not uniformly high.	Require expert verification for radiological-anatomy outputs and reassess systems after model/mode changes rather than relying on prior validation.	Individual educator	No	No	Yes	High	5	5	5	5	PubMed abstract: overall accuracy 47.4%-85.7%; ChatGPT-5 stability fell from substantial to moderate agreement in Thinking mode.	Anatomical fidelity	Excellent evidence for version/mode-specific governance and revalidation.
41	Pasli Beser, 2026	Paslı B, Günenç Beşer C. Is artificial intelligence getting better at anatomy? A two-year review of ChatGPT's free public versions. Anatomical Sciences Education. 2026 May 14. Online ahead of print. doi:10.1002/ase.70255.	2026	Türkiye	https://doi.org/10.1002/ase.70255	Original empirical research	Online first	Yes	Anatomy education	Yes	Direct anatomy evidence	Large language model	Learning support	Assessment content	3. Anatomical fidelity & source verification	Anatomical accuracy improves across model generations but residual errors persist and error types remain predominantly informational.	Need for version-specific validation and continued checking despite apparent technological improvement.	Outdated governance decisions may assume a model remains equally reliable after updates; learners may over-trust improved but imperfect systems.	Learner	Using the same 120 anatomy MCQs over ~2 years, correct-response rates increased from 45.8% (ChatGPT-3.5) to 73.3% (4o) and 86.7% (5), but informational errors remained the predominant error type.	Revalidate after major model/version changes and retain human verification for substantive anatomy content even when benchmark accuracy improves.	Individual educator	No	Partial/indirect	Yes	Moderate	5	5	5	5	PubMed abstract: 45.8% → 73.3% → 86.7%; informational errors 72.3%-81.25% of incorrect responses.	Anatomical fidelity	Strong evidence against static one-time approval of a model.
42	Ulkir Pasli 2026	Ülkir M, Paslı B. Reference Hallucination, Citation Reliability, and Readability of Large Language Models in Anatomy-Related Question Answering. Clinical Anatomy. 2026 Jul 15. Online ahead of print. doi:10.1002/ca.70187.	2026	Türkiye	https://doi.org/10.1002/ca.70187	Original empirical research	Online first	Yes	Anatomy education/scholarly reference use	Yes	Direct anatomy evidence	Large language model	Teaching explanation	Public text	3. Anatomical fidelity & source verification	LLMs generate substantial levels of fabricated or unreliable anatomy-related references even when responses appear plausible.	Bibliographic integrity, source verification, and academic trust.	Fabricated citations, false PMIDs, and unsupported references may enter teaching materials, assignments, or scholarship.	Multiple stakeholders	Across 1,800 references generated for 120 anatomy questions, reference hallucination rates were 23.2%-47.5%; PMID accuracy was only 25.1%-57.6%, and fully supported citations ranged from 41.0% to 67.2%.	Independently verify that every AI-generated citation exists and that the cited source actually supports the claim before educational or scholarly use.	Individual educator	No	No	Yes	High	5	5	5	5	PubMed abstract: hallucination 23.2%-47.5%; PMID accuracy 25.1%-57.6%; authors state human verification remains essential.	Anatomical fidelity	One of the strongest direct sources for a mandatory citation-verification control.
43	Sarantopoulos, 2026	Sarantopoulos A, Pana ZD, Larentzakis A, Kondylis S, Maina A, Ziogas N, Ntourakis D. ChatGPT-4.0 and Medical Students: A Recognition-Gated Comparative Evaluation on Image-Based Medical Examinations. Journal of Medical Education and Curricular Development. 2026;13:23821205261449387. doi:10.1177/23821205261449387.	2026	Cyprus	https://doi.org/10.1177/23821205261449387	Original empirical research	Published	Yes	Medical education/image-based anatomy assessment	Partial/indirect	Transferable HPE evidence	Multimodal generative model	Imaging/radiological anatomy	Medical image	3. Anatomical fidelity & source verification	Vision-capable LLM performance can be constrained by failure to recognize images before downstream reasoning is even attempted.	Recognition failure as a distinct safety gate in image-dependent educational use.	An apparently capable model may provide no reliable reasoning when the image itself is not correctly recognized.	Learner	In image-based examinations, ChatGPT-4.0 significantly underperformed students in Anatomy; when image-recognition failures were scored as incorrect, global anatomy accuracy was 17.3%.	Use a recognition gate before accepting downstream reasoning from multimodal AI; failed/uncertain image recognition should trigger rejection or human escalation.	Individual educator	No	Yes	Yes	High	5	5	5	5	PubMed/PMC abstract: global end-to-end anatomy accuracy 17.3% when image-recognition failures counted as incorrect.	Anatomical fidelity	Very useful for separating image-recognition verification from reasoning verification.
45	Bae, 2025 Anatomy Images	Bae JS, Kim GY, Kim HJ, Han SH, Youn KH. Comparison of anatomy image generation capability in AI image generation models. J Vis Commun Med. 2025;48(2):44-51. doi:10.1080/17453054.2025.2504491.	2025	Republic of Korea	https://doi.org/10.1080/17453054.2025.2504491	Original empirical research	Published	Yes	Anatomy/biomedical illustration	Yes	Direct anatomy evidence	Text-to-image	Anatomical image generation	AI-generated image	3. Anatomical fidelity & source verification	General-purpose image generators can produce visually plausible anatomy while remaining structurally inaccurate, and performance changes across model generations.	Educational images may be mistaken for authoritative anatomy despite model-specific structural errors.	Incorrect spatial or structural mental models; propagation of visually persuasive but false anatomy.	Learner	Compared DALL-E 2, Midjourney 5 and Stable Diffusion 1.5 across bones, organs and muscles, with later comparison using Midjourney 6 and DALL-E 3. Despite improvement, the authors concluded that current systems cannot replace professional medical illustrators.	Require expert anatomical review before learner-facing use; assess structure rather than visual polish alone; re-audit after major model/version changes.	Individual educator	No	Partial/indirect	Direct	High	5	5	5	5	PubMed/Taylor & Francis abstract; bones, organs and muscles; model-generation comparison.	Anatomical fidelity	S4 addition; direct anatomy-image evidence complementing Eldesoqui et al.
46	Kumar, 2024 Medical Illustrations	Kumar A, Burr P, Young TM. Using AI Text-to-Image Generation to Create Novel Illustrations for Medical Education: Current Limitations as Illustrated by Hypothyroidism and Horner Syndrome. JMIR Med Educ. 2024;10:e52155. doi:10.2196/52155.	2024	United Kingdom	https://doi.org/10.2196/52155	Original empirical research	Published	Yes	Medical education/medical illustration	Partial/indirect	Transferable HPE evidence	Text-to-image	Resource generation	AI-generated image	3. Anatomical fidelity & source verification	Text-to-image systems may generate convincing clinical illustrations while failing to reproduce diagnostically important physical signs accurately.	Synthetic clinical imagery can obscure the difference between illustration and evidence-based visual exemplar.	Learners may encode incorrect morphology or clinical signs from plausible-looking generated images.	Learner	Using hypothyroidism and Horner syndrome as examples, the study demonstrated practical limitations of widely available text-to-image tools for educational medical illustrations.	Use AI images only after expert clinical/anatomical validation; define whether the image is illustrative or intended as a ground-truth exemplar; reject outputs that fail the educational objective.	Individual educator	No	No	Direct	High	4	5	4	4.3	JMIR article/PMID 38386400; limitations demonstrated using hypothyroidism and Horner syndrome.	Anatomical fidelity	S4 addition; transferable visual-fidelity evidence rather than anatomy-specific structural study.
47	Currie, 2024 Medical Students	Currie G, Currie J, Anderson S, Hewis J. Gender bias in generative artificial intelligence text-to-image depiction of medical students. Health Educ J. 2024;83(7):732-746. doi:10.1177/00178969241274621.	2024	Australia	https://doi.org/10.1177/00178969241274621	Original empirical research	Published	Yes	Medical education/representation	No	Transferable HPE evidence	Text-to-image	Resource generation	AI-generated image	4. Variation, bias & representation	Generated medical-student imagery can systematically misrepresent gender and skin-tone distributions.	Repeated learner exposure may normalize distorted prototypes of who belongs in medicine.	Stereotype reinforcement, exclusion, and biased professional identity formation.	Learner	Across 448 depicted medical students, 58.8% were coded as men and 92.0% as light skin tone; no characters were coded with dark skin tone. Gender distribution differed significantly from actual Australian medical-student demographics.	Audit collections of generated images against an explicit, purpose-appropriate representation benchmark; document prompts and sampling; curate sets rather than accepting default outputs.	Individual educator	No	No	Partial/indirect	Moderate	4	5	5	4.6	Results section: N=448; 58.8% men; 92.0% light skin tone; 0% dark skin tone.	Bias/representation	S4 addition; demonstrates that bias often emerges across image sets, not only single images.
48	Bui, 2026 Medical Students	Bui M, Yi PH, Onuh I, Kuckelman I, Ross AB. Portrayal of Medical Students in Artificial Intelligence-Generated Images. WMJ. 2026;125(1):158-161. PMID:41980156.	2026	United States	https://pubmed.ncbi.nlm.nih.gov/41980156/	Original empirical research	Published	Yes	Medical education/representation	No	Transferable HPE evidence	Text-to-image	Resource generation	AI-generated image	4. Variation, bias & representation	A single neutral prompt can produce demographic distributions and role/attire associations that diverge from the real learner population.	Bias may operate through both who is depicted and how different groups are visually framed.	Stereotype reinforcement and inaccurate representation of the learner population.	Learner	Among 300 DALL-E images prompted with 'medical student,' 76% were coded female and 74% White; no Native American/Alaska Native or Native Hawaiian/Pacific Islander students were represented, and attire differed by perceived race/ethnicity.	Benchmark generated sets against relevant real-world demographics when representation is educationally material; audit both demographic frequency and role/status cues.	Individual educator	No	No	Partial/indirect	Moderate	4	5	5	4.6	PubMed/WMJ abstract: 300 images; 76% female; 74% White; absent groups and attire differences.	Bias/representation	S4 addition; useful counterpoint showing that bias direction can differ across model/prompt/context.
49	Shaari, 2025 Otolaryngology	Shaari AL, Saad AM, Patel AM, Filimonov A. Representation of Demographics in Otolaryngology by Artificial Intelligence Text-to-Image Platforms. Laryngoscope Investig Otolaryngol. 2025;10(3):e70152. doi:10.1002/lio2.70152.	2025	United States	https://doi.org/10.1002/lio2.70152	Original empirical research	Published	Yes	Surgical education/professional representation	No	Transferable HPE evidence	Text-to-image	Resource generation	AI-generated image	4. Variation, bias & representation	Demographic distortion varies across generators and across academic rank or subspecialty, so 'AI image bias' is not a single fixed property.	Generated professional imagery may amplify status-linked stereotypes and underrepresentation.	Marginalization, stereotype reinforcement, and distorted professional-role expectations.	Learner	Across 1,740 generated otolaryngologist portraits, 88% were coded White and 88% male; department chairs and professors were especially skewed. DALL-E 3, Midjourney and Runway differed significantly.	Audit by model and use case; assess rank/status cues as well as demographics; do not assume one platform's performance generalizes to another.	Individual educator	No	No	Partial/indirect	Moderate	4	5	5	4.6	PubMed/PMC: 1,740 portraits; 88% White, 88% male; model and rank differences.	Bias/representation	S4 addition; supports model-specific and context-specific visual auditing.
50	Currie, 2025 Imaging Bias	Currie G, Hewis J, Hawk E, Rohren E. Gender and Ethnicity Bias of Text-to-Image Generative Artificial Intelligence in Medical Imaging, Part 2: Analysis of DALL-E 3. J Nucl Med Technol. 2025;53(2):162-168. doi:10.2967/jnmt.124.268359.	2025	Australia/United States	https://doi.org/10.2967/jnmt.124.268359	Original empirical research	Published	Yes	Medical imaging professions/representation	No	Transferable HPE evidence	Text-to-image	Resource generation	AI-generated image	4. Variation, bias & representation	Even a newer text-to-image model may overrepresent dominant demographic groups and omit disability representation across medical professions.	Educational and professional imagery may reproduce inequity while appearing polished and neutral.	Narrow professional prototypes and exclusion of underrepresented groups, including disability.	Learner	Across 524 depicted medical-imaging professionals generated with DALL-E 3, 57.4% were male and 91.2% had light skin tone; no person with a disability was represented.	Include disability and other relevant dimensions in representation audits; compare outputs with appropriate workforce or curricular benchmarks; curate image sets before use.	Individual educator	No	No	Partial/indirect	Moderate	4	5	5	4.6	PubMed abstract: n=524; 57.4% male; 91.2% light skin; no disability representation.	Bias/representation	S4 addition; broadens representation beyond race/gender and supports explicit audit dimensions.
51	Ali, 2024 Surgeon Representation	Ali R, Tang OY, Connolly ID, et al. Demographic Representation in 3 Leading Artificial Intelligence Text-to-Image Generators. JAMA Surg. 2024;159(1):87-95. doi:10.1001/jamasurg.2023.5695.	2024	United States	https://doi.org/10.1001/jamasurg.2023.5695	Original empirical research	Published	Yes	Surgery/professional representation	No	Transferable HPE evidence	Text-to-image	Resource generation	AI-generated image	4. Variation, bias & representation	Text-to-image models can amplify rather than merely mirror existing demographic disparities; prompt changes may improve one dimension without correcting another.	Bias mitigation based on prompting alone may create a false sense of correction.	Amplification of stereotypes and misleading visual norms about the profession.	Learner	Across 2,400 surgeon images plus 1,200 geographically prompted images, two models depicted more than 98% of surgeons as White and male; geographic prompting increased non-White representation but did not correct female underrepresentation.	Use external benchmarks and post-generation auditing/curation rather than relying on prompt engineering alone; document model, prompt and sampling approach.	Individual educator	No	No	Partial/indirect	Moderate	4	5	5	4.6	JAMA Surgery key points/conclusion: >98% White and male in two models; geography prompt affected race but not gender.	Bias/representation	S4 addition; strong evidence that prompt-level mitigation is incomplete and model-dependent.
52	Senft Everson, 2025 Cancer Survivor	Senft Everson N, Gaysynsky A, Iles IA, Schrader KE, Chou W-YS. What does an AI-generated 'cancer survivor' look like? An analysis of images generated by text-to-image tools. J Cancer Surviv. 2025. doi:10.1007/s11764-025-01760-1.	2025	United States	https://doi.org/10.1007/s11764-025-01760-1	Original empirical research	Published	Yes	Patient-facing medical imagery/representation	No	Transferable HPE evidence	Text-to-image	Resource generation	AI-generated image	4. Variation, bias & representation	Generated imagery may encode narrow cultural narratives as well as demographic skew, even when no demographic characteristics are specified in the prompt.	Visual stereotypes can shape expectations about disease, bodies and lived experience.	Narrow or misleading mental models of patients and disease experience; representational harm.	Patient/public	In 160 DALL-E/Stable Diffusion images, cancer survivors were commonly portrayed as White (80%), feminine (80%), young (51%), happy (69%) and healthy (80%), with recurrent visual tropes such as pink and cancer ribbons.	Audit narrative and contextual tropes in addition to demographic counts; align representation checks with the intended educational purpose and patient population.	Individual educator	No	No	Partial/indirect	Moderate	3	5	4	3.9	Springer/PMC results: 160 images; recurrent demographic and illness-narrative stereotypes.	Bias/representation	S4 addition; useful for contextual/role bias, but indirect for anatomy-specific governance.
53	Lopiano, 2026	Lopiano S, Cohen ES, Holan G, Grachan JJ. Prompting the Question: Evaluating ChatGPT-Generated Gross Anatomy and Embryology Questions for Accuracy and NBME Item-Writing Style. Cureus. 2026;18(4):e107397. doi:10.7759/cureus.107397.	2026	United States	https://doi.org/10.7759/cureus.107397	Original empirical research	Published	Yes	Gross anatomy/embryology assessment	Yes	Direct anatomy evidence	Large language model	Question generation	Assessment content	5. Assessment validity & integrity	Prompt refinement can improve higher-order cognitive demand in AI-generated anatomy questions, but may reduce factual accuracy; stylistic conformity does not guarantee valid content.	AI-generated items may contain factual errors, multiple correct answers, low cognitive demand, or item-writing flaws despite appearing NBME-like.	Invalid or misleading assessment items; distorted inference about learner competence; inefficient faculty review.	Learner	Across 510 ChatGPT-4-generated gross anatomy/embryology MCQs, initial prompts yielded 80.0% accuracy but only 7.7% higher-order questions. Modified prompts increased higher-order questions to 41.0% while accuracy fell to 70.5%. Authors concluded that item quality remained variable and required additional review.	Require expert content review plus item-writing review; verify single-best-answer status; assess cognitive level explicitly; do not treat prompt sophistication as a substitute for quality assurance.	Assessment committee	No	Yes	Direct	High	5	5	5	5	PubMed/PMC/Cureus: 510 generated questions; 80.0%→70.5% accuracy and 7.7%→41.0% higher-order after prompt modification.	Assessment	S5 addition; strong direct evidence of an accuracy-versus-cognitive-complexity tradeoff in anatomy item generation.
54	Kiyak Peker, 2025	Kıyak YS, Soylu A, Coşkun Ö, Budakoğlu II, Peker TV. Can ChatGPT Generate Acceptable Case-Based Multiple-Choice Questions for Medical School Anatomy Exams? A Pilot Study on Item Difficulty and Discrimination. Clinical Anatomy. 2025;38:505-510. doi:10.1002/ca.24271.	2025	Türkiye	https://doi.org/10.1002/ca.24271	Original empirical research	Published	Yes	Medical school anatomy assessment	Yes	Direct anatomy evidence	Large language model	Question generation	Assessment content	5. Assessment validity & integrity	After expert review and departmental approval, AI-generated case-based anatomy MCQs can show acceptable psychometric characteristics in real examinations.	Promising post-hoc psychometrics can be misinterpreted as evidence that unreviewed AI items are safe for direct use.	Poor-quality items entering examinations if pre-use expert review and item analysis are bypassed.	Learner	ChatGPT-generated case-based anatomy MCQs were expert-reviewed, approved by the department, and administered to 502 second-year medical students. Discrimination indices ranged from 0.29 to 0.54, while item difficulty ranged from 0.41 to 0.89; authors emphasized that human expertise remained crucial for review and refinement.	Use AI as an item-drafting tool within the existing assessment-governance process; require expert approval before deployment and post-hoc psychometric review after use.	Assessment committee	No	Yes	Direct	High	5	5	5	5	Clinical Anatomy/PubMed: n=502; discrimination indices 0.29-0.54; item difficulty 0.41-0.89; expert review preceded administration.	Assessment	S5 addition; direct evidence that acceptable item statistics were observed after human expert review and departmental approval, not after autonomous item deployment. Author list corrected against the published record.
55	Tekin, 2025	Tekin A, Karamus NF, Çolak T. Anatomy exam model for the circulatory and respiratory systems using GPT-4: a medical school study. Surg Radiol Anat. 2025;47:158. doi:10.1007/s00276-025-03667-z.	2025	Türkiye	https://doi.org/10.1007/s00276-025-03667-z	Original empirical research	Published	Yes	Anatomy assessment/medical school	Yes	Direct anatomy evidence	Large language model	Question generation	Assessment content	5. Assessment validity & integrity	AI-generated anatomy items can produce workable test-level psychometrics while still failing to deliver the intended cognitive level reliably.	Psychometric acceptability alone may conceal construct underrepresentation or misclassification of cognitive demand.	Assessment may over-sample recall and under-assess higher-order anatomical reasoning.	Learner	GPT-4 generated 240 anatomy MCQs; 30 were administered to 280 students. The test KR-20 was 0.737 and mean difficulty 0.5012, but expert anatomists agreed with GPT-4's Bloom-level classification for only 26.6% of questions. Authors concluded that higher-order cognitive processes were insufficiently assessed.	QA should evaluate construct/cognitive-level alignment in addition to item difficulty and discrimination; blueprint items against learning outcomes and independently classify cognitive demand.	Assessment committee	No	Yes	Direct	High	5	5	5	5	Springer abstract/full page: 240 generated items, 30 administered to 280 students; KR-20 0.737; expert/GPT taxonomy match 26.6%.	Assessment	S5 addition; particularly strong for separating psychometric performance from construct/cognitive validity.
56	Bernard, 2024 OSPE	Bernard J, Sonnadara R, Saraco AN, Mitchell JP, Bak AB, Bayer I, Wainman BC. Automated grading of anatomical objective structured practical examinations using decision trees: An artificial intelligence approach. Anat Sci Educ. 2024;17:967-978. doi:10.1002/ase.2305.	2024	Canada	https://doi.org/10.1002/ase.2305	Original empirical research	Published	Yes	Anatomy OSPE grading	Yes	Direct anatomy evidence	Conventional AI (foundational only)	Scoring/grading	Student work/data	5. Assessment validity & integrity	Task-specific AI can grade short-answer anatomy OSPE responses with high agreement against human marking when developed and validated on local assessment data.	High average grading accuracy may not generalize to new stations, cohorts, answer variants, or consequential use without local validation and error monitoring.	Incorrect marks, unfairness, missed legitimate answer variants, and false confidence in automated scoring.	Learner	Decision trees trained on 90% of responses from a 54-question final anatomy OSPE and tested on the remaining 10% achieved an average grading accuracy of 94.49% compared with staff/faculty marking.	Before automated scoring is used beyond practice, validate locally against expert marking; define acceptable error thresholds, ambiguous-answer handling, human override, and ongoing monitoring.	Assessment committee	No	Yes	Direct	High	5	5	5	5	Anatomical Sciences Education abstract: 54 OSPE questions; 90/10 training-validation design; mean grading accuracy 94.49%.	Assessment	S5 addition; foundational conventional-AI evidence directly relevant to governance of automated anatomy scoring.
57	Mezil, 2026 AI OSPE	Mezil Y, Yu P, Rahimpour L, Wang T, Rajyam S, Varambally M, et al. Harnessing artificial intelligence for automatic feedback in a virtual anatomy study tool: A Q-methodology study. Anat Sci Educ. 2026;19(5):684-694. doi:10.1002/ase.70206.	2026	Saudi Arabia/Canada	https://doi.org/10.1002/ase.70206	Original empirical research	Published	Yes	Anatomy OSPE practice/feedback	Yes	Direct anatomy evidence	Conventional AI (foundational only)	Feedback	Student work/data	5. Assessment validity & integrity	AI can support low-stakes anatomy OSPE practice by grading responses and providing immediate personalized feedback, but learner-acceptance evidence is not equivalent to evidence for high-stakes grading validity.	Positive learner perceptions may be overextended to claims about scoring reliability or suitability for summative use.	Over-reliance on automated practice feedback; inappropriate migration from formative tutoring to consequential grading.	Learner	The AI OSPE provided practice stations, automated grading and immediate personalized feedback. Q-methodology in first-year Occupational Therapy students identified learner preference profiles and areas for tool improvement; the study focused on perceptions rather than validating autonomous summative grading.	Keep automated feedback clearly formative unless scoring validity has been separately demonstrated; display expert-defined answers, confidence/uncertainty where available, and a route for users to flag disputed grading.	Course/module lead	No	Yes	Direct	Moderate	5	5	4	4.7	PubMed/Wiley abstract and figures: AI OSPE used pre-determined expert answers, automated grading and immediate feedback; Q-methodology assessed perceptions.	Assessment	S5 addition; strong evidence for separating low-stakes practice feedback from high-stakes assessment decisions.
58	Al Mashhadani, 2026 Feedback	Al-Mashhadani MM, Ajaz F, Guraya SS, Ennab F. Use of large language models for providing automated feedback in medical imaging education: a systematic review. Front Med (Lausanne). 2026;13:1803921. doi:10.3389/fmed.2026.1803921.	2026	United Arab Emirates	https://doi.org/10.3389/fmed.2026.1803921	Systematic review	Published	Yes	Medical imaging education/automated feedback	No	Transferable HPE evidence	Large language model	Feedback	Student work/data	5. Assessment validity & integrity	Automated LLM feedback can improve selected learning outcomes and efficiency in imaging education, but evidence remains preliminary, model agreement with experts varies, and learners tend to prefer hybrid human-AI feedback.	Technical feasibility may be mistaken for proven educational effectiveness or readiness for autonomous evaluative decisions.	Misleading feedback; propagation of model errors; replacement of expert formative judgment before evidence is mature.	Learner	The systematic review identified 7 studies from 1,003 records. Reported benefits included improved report quality, diagnostic accuracy and discrepancy detection; learners generally preferred hybrid human-AI feedback, and model agreement with expert consensus was variable. Authors concluded that human expert oversight remains essential.	Use automated feedback as supportive rather than substitutive; validate against expert standards, retain human escalation, and avoid consequential use until stronger evidence exists.	Course/module lead	No	Partial/indirect	Partial/indirect	Moderate	4	5	5	4.6	PubMed abstract: 7 included studies; hybrid feedback preferred; variable agreement with experts; human oversight essential.	Assessment	S5 addition; transferable evidence for feedback governance where anatomy-specific evidence remains limited.
59	Ahmed, 2025 SBA Quality	Ahmed A, Kerr E, O'Malley A. Quality assurance and validity of AI-generated single best answer questions. BMC Med Educ. 2025;25:300. doi:10.1186/s12909-025-06881-w.	2025	United Kingdom	https://doi.org/10.1186/s12909-025-06881-w	Original empirical research	Published	Yes	Medical education assessment	No	Transferable HPE evidence	Large language model	Question generation	Assessment content	5. Assessment validity & integrity	AI-generated examination items can perform comparably to human-authored items after robust pre-use quality assurance, but a substantial fraction may need rejection before they are safe to deploy.	Post-hoc equivalence of accepted items can obscure the high pre-use rejection burden and the importance of expert screening.	Erroneous or curriculum-misaligned questions entering assessment banks if automated generation bypasses faculty QA.	Learner	GPT-4 generated 220 SBA questions. Expert QA found 69% fit for inclusion with little/no modification and rejected 31% because of factual inaccuracies or non-alignment with learning. Accepted AI items showed no significant difference from human-authored items in facility or discrimination.	Require structured expert QA and explicit reject criteria before assessment use; for summative items, retain standard panel review and post-hoc item analysis.	Assessment committee	No	Yes	Partial/indirect	High	4	5	5	4.6	BMC full text: 220 generated SBAs; 31% rejected pre-use; accepted items comparable with human items on facility/discrimination.	Assessment	S5 addition; transferable but methodologically strong evidence for a mandatory pre-use QA gate.
60	Meo, 2024 OSPE Bard	Meo SA, AbuKhalaf AA, Meo MZS, Meo MO, Ayub R, ElToukhy RA, Usmani AM, Hajjar WM. Role of artificial intelligence (Google Bard) in morphological, histopathological, and radiological image identifications: Objective Structured Practical Examination (OSPE) type-based performance. Saudi Med J. 2024;45(5):531-536. doi:10.15537/smj.2024.45.5.20240141.	2024	Saudi Arabia	https://doi.org/10.15537/smj.2024.45.5.20240141	Original empirical research	Published	Yes	OSPE-type image identification/medical sciences	Partial/indirect	Transferable HPE evidence	Multimodal generative model	Practical/OSPE	Medical image	5. Assessment validity & integrity	General-purpose multimodal AI performance on OSPE-style image identification is highly variable across anatomical, histological and radiological content.	Apparent competence on some image types may create false confidence for practical-examination support or automated scoring.	Incorrect identification or interpretation in image-dependent practical assessment.	Learner	Google Bard answered 34 of 60 OSPE-type image-identification tasks correctly overall (56.7%), with performance ranging from 28.6% for kidney images to 90% for bone/radiological images.	Do not use unvalidated general-purpose multimodal AI as an autonomous judge of anatomy practical performance; require task-specific validation and expert fallback.	Assessment committee	No	Yes	Direct	High	4	5	4	4.3	PubMed abstract: overall 34/60 (56.7%); performance varied markedly by image/content category.	Assessment	S5 addition; supports high caution for image-dependent practical assessment and reinforces S3 recognition-gate findings.
61	AAMC, 2025 Responsible AI	Association of American Medical Colleges. Principles for the Responsible Use of Artificial Intelligence in and for Medical Education. Version 2.0 completed July 31, 2025.	2025	United States	https://www.aamc.org/about-us/mission-areas/medical-education/principles-ai-use	Professional guideline	Guidance/policy	No	Medical education governance	No	Professional/normative guidance	General generative AI	Institutional governance	Institutional content	8. Institutional authorization, monitoring & incident response	Medical-education AI governance should operate across individual, institutional and community levels, retain human judgment, protect privacy, document AI use, and frequently evaluate tools in their intended context.	Institutions can normalize AI use without specifying accountability, disclosure, privacy, monitoring, or escalation requirements.	Unsafe or inequitable implementation, privacy breaches, unclear responsibility, and persistence of poorly performing tools.	Institution	AAMC version 2.0 sets seven principles: human-centered focus; ethical and transparent use; equal access; training; interdisciplinary collaboration; data privacy; and monitor/evaluate. It recommends documented review cycles, performance thresholds, escalation procedures, and structured monitoring for tools affecting assessment or clinical education.	Create anatomy-specific local policy that names approved uses, required disclosures, data restrictions, review frequency, performance thresholds, escalation routes, and responsible roles.	Institution/medical school	No	Yes	Partial/indirect	High	5	5	5	5	AAMC principles page; v2.0 31 July 2025. Monitor & Evaluate section recommends review cycles, thresholds and escalation for assessment/clinical education.	Institutional governance	S6 addition; highly relevant medical-education governance source and direct bridge from broad HPE principles to anatomy-specific policy.
62	Masters, 2023 AMEE158	Masters K. Ethical use of Artificial Intelligence in Health Professions Education: AMEE Guide No. 158. Med Teach. 2023;45(6):574-584. doi:10.1080/0142159X.2023.2186203.	2023	Oman	https://doi.org/10.1080/0142159X.2023.2186203	Professional guideline	Published	Yes	Health professions education ethics	No	Transferable HPE evidence	General generative AI	Institutional governance	Student work/data	7. Human oversight & accountability	HPE AI use raises interconnected ethical issues involving data gathering, privacy, consent, ownership, security, bias, transparency, responsibility, autonomy and beneficence.	Technical adoption without ethical governance can compromise educator/learner autonomy and obscure responsibility for AI-supported decisions.	Privacy loss, biased educational decisions, weak consent, unclear accountability, and erosion of professional autonomy.	Multiple stakeholders	AMEE Guide 158 frames AI ethics from the perspective of HPE teachers and administrators and calls for proactive institutional preparation for data, privacy, bias, transparency, responsibility and autonomy concerns.	Requires explicit responsibility assignments, privacy/data rules, institutional ethical oversight, stakeholder education, and safeguards that preserve human professional judgment.	Institution/medical school	No	Partial/indirect	Partial/indirect	High	4	5	5	4.6	Medical Teacher full text/PubMed PMID 36912253; ethics topics include privacy, consent, ownership, bias, transparency, responsibility and autonomy.	Accountability/oversight	S6 addition; foundational HPE ethics source. Normative recommendations should be distinguished from empirical validation.
63	NIST, 2024 GenAIRMF	Autio C, Schwartz R, Dunietz J, et al. Artificial Intelligence Risk Management Framework: Generative Artificial Intelligence Profile. NIST AI 600-1. National Institute of Standards and Technology; 2024. doi:10.6028/NIST.AI.600-1.	2024	United States	https://doi.org/10.6028/NIST.AI.600-1	Policy/framework document	Published	No	Cross-sector generative-AI risk governance	No	Professional/normative guidance	General generative AI	Institutional governance	Institutional content	8. Institutional authorization, monitoring & incident response	Generative-AI risk management should be use-case specific and lifecycle based, with governance, content provenance, pre-deployment testing, human oversight, documentation, continuous monitoring, third-party risk management and incident disclosure.	Organizations may deploy external GenAI tools without maintaining inventories, provenance records, review histories, monitoring or incident-response processes.	Undetected model drift or failure, repeated unsafe use, untraceable content, third-party exposure, and inability to investigate or correct incidents.	Institution	NIST AI 600-1 organizes GenAI risk actions around GOVERN, MAP, MEASURE and MANAGE. It explicitly highlights Governance, Content Provenance, Pre-deployment Testing and Incident Disclosure; recommends documenting human oversight roles and model/version information, monitoring outcomes, maintaining change history, approved-provider lists, and processes for incident response.	Maintain an anatomy-AI use inventory; document tool/model/version, use case, data/input class, responsible owner and review date; pretest higher-risk uses; monitor performance; record overrides/errors; define withdrawal, correction and incident-escalation procedures.	Institution/medical school	Yes	Yes	Partial/indirect	High	5	5	5	5	NIST AI 600-1 pp. 4-5, 19-24, 27-28, 31-43, 54-56: governance, provenance, monitoring, human oversight, approved providers and incident disclosure.	Institutional governance	S6 addition; strongest operational lifecycle source for monitoring, third-party governance, documentation and incident response.
64	Lekadir, 2025 FUTUREAI	Lekadir K, Frangi AF, Porras AR, et al. FUTURE-AI: international consensus guideline for trustworthy and deployable artificial intelligence in healthcare. BMJ. 2025;388:e081554. doi:10.1136/bmj-2024-081554.	2025	International	https://doi.org/10.1136/bmj-2024-081554	Consensus statement	Published	Yes	Healthcare AI governance	No	Transferable HPE evidence	Conventional AI (foundational only)	Institutional governance	Institutional content	8. Institutional authorization, monitoring & incident response	Trustworthy AI requires lifecycle governance rather than one-time validation, with fairness, universality, traceability, usability, robustness and explainability supported by continuous risk assessment and multistakeholder oversight.	A tool that performs acceptably once may be treated as permanently trustworthy despite changes in model, users, context or risk.	Performance drift, inequity, context failure, and deployment beyond validated conditions.	Institution	The international FUTURE-AI consortium of 117 experts from 50 countries developed 30 best practices spanning design, development, validation, regulation, deployment and monitoring, explicitly emphasizing continuous risk assessment and mitigation.	Treat approval as conditional and time-limited; define validated context and intended users; reassess after substantive tool/version/workflow changes; include domain experts and affected stakeholders in higher-risk review.	Institution/medical school	No	Partial/indirect	Partial/indirect	High	4	5	5	4.6	BMJ 2025;388:e081554: six principles, 30 best practices, lifecycle coverage and continuous risk assessment.	Institutional governance	S6 addition; clinical rather than educational, but highly transferable for lifecycle monitoring and conditional authorization.
65	Gin, 2025 Entrustment	Gin BC, O'Sullivan PS, Hauer KE, Abdulnour RE, Mackenzie M, ten Cate O, Boscardin CK. Entrustment and EPAs for Artificial Intelligence (AI): A Framework to Safeguard the Use of AI in Health Professions Education. Acad Med. 2025;100(3):264-272. doi:10.1097/ACM.0000000000005930.	2025	United States/The Netherlands	https://doi.org/10.1097/ACM.0000000000005930	Policy/framework document	Published	Yes	Health professions education governance	No	Transferable HPE evidence	General generative AI	Institutional governance	Institutional content	7. Human oversight & accountability	AI participation in HPE should be task-specific and explicitly entrusted rather than globally approved; some consequential tasks may never be appropriate for autonomous AI.	Binary 'AI allowed/not allowed' policies fail to define the degree of supervision needed for different tasks and consequences.	Overdelegation, automation bias, deskilling, and inappropriate autonomous decisions affecting learners.	Multiple stakeholders	Gin et al. adapt entrustment to AI using ability, integrity and benevolence and propose explicit levels of AI participation/supervision. They note that some tasks, such as summative promotion/remediation decisions, may never warrant unsupervised AI.	Define permitted AI role per anatomy task: observe/support, draft with review, act with close supervision, or restricted/non-delegable. Tie required human supervision to task consequence and evidence of trustworthiness.	Course/module lead	No	Yes	Partial/indirect	High	5	5	5	5	Academic Medicine 2025;100:264-272; entrustment scale and examples of tasks that may remain permanently supervised/non-delegable.	Accountability/oversight	S6 addition; especially useful for converting our risk tiers into explicit permissible degrees of AI autonomy.
66	Izquierdo Condoy, 2026 FACETS	Izquierdo-Condoy JS, Arias-Intriago M, Montero Corrales L, Ortiz-Prado E. Artificial Intelligence in Medical Education: Transformative Potential, Current Applications, and Future Implications. JMIR Medical Education. 2026;12:e77127. doi:10.2196/77127.	2026	International	https://doi.org/10.2196/77127	Narrative review	Published	Yes	Medical education implementation	No	Transferable HPE evidence	General generative AI	Institutional governance	Institutional content	8. Institutional authorization, monitoring & incident response	AI implementation should be pedagogically contextualized and institutionally governed, with predeployment audit, privacy-preserving workflows, human oversight for high-impact uses and ongoing psychometric/equity monitoring.	Institutions may evaluate only technical capability while neglecting educational purpose, privacy workflow, equity impact and postdeployment performance.	Pedagogically inappropriate use, privacy leakage, inequity, and uncontrolled performance degradation.	Institution	The 2026 review proposes the FACETS implementation lens and recommends human oversight for high-impact uses, predeployment bias/drift/robustness audits, privacy-preserving workflows, secure logging, and ongoing monitoring tied to educational outcomes.	Before anatomy-AI deployment, document educational purpose/context, privacy pathway and supervision level; for high-impact uses require a predeployment audit plus ongoing educational-performance and equity monitoring.	Institution/medical school	No	Yes	Partial/indirect	High	4	4	5	4.3	JMIR Med Educ 2026;12:e77127: FACETS framework; recommendations include human oversight for high-impact uses, predeployment audits, privacy-preserving workflows, secure logging, and ongoing psychometric/equity monitoring.	Institutional governance	S6 addition; useful implementation synthesis but should not be treated as consensus guidance. Author attribution corrected against the final JMIR publication.
67	OECD, 2024 AI Principles	Organisation for Economic Co-operation and Development. OECD AI Principles. Adopted 2019; updated 2024.	2024	International	https://www.oecd.org/en/topics/ai-principles.html	Policy/framework document	Guidance/policy	No	Cross-sector AI governance	No	Professional/normative guidance	General generative AI	Institutional governance	Institutional content	7. Human oversight & accountability	AI governance should be human-centered, transparent, traceable, robust, safe and accountable, with systematic risk management throughout the lifecycle and mechanisms to override, repair or decommission systems exhibiting undesired behavior.	Responsibility may become diffuse across educators, institutions, vendors and systems unless role-based accountability and redress mechanisms are explicit.	Unchallengeable decisions, persistence of harmful outputs, unclear accountability and weak response to known failure.	Multiple stakeholders	Updated OECD principles explicitly address generative AI and call for human agency/oversight, transparency and responsible disclosure, traceability, continuous lifecycle risk management, accountability according to actors' roles, and mechanisms to override, repair or decommission unsafe systems.	Assign role-based accountability; provide routes to challenge AI-supported outcomes; preserve human override; define correction/withdrawal/decommission criteria when risk controls fail.	Multiple actors	No	Partial/indirect	Partial/indirect	High	4	5	5	4.6	OECD AI Principles updated May 2024: human agency/oversight, transparency, traceability, robustness/safety and accountability throughout lifecycle.	Accountability/oversight	S6 addition; broad normative source supporting override, redress, accountability and lifecycle risk management.
68	UNESCO, 2023 GenAI	Miao F, Holmes W. Guidance for Generative AI in Education and Research. Paris: UNESCO; 2023 (updated online 2026).	2023	Global	https://www.unesco.org/en/articles/guidance-generative-ai-education-and-research	Professional guideline	Guidance/policy	No	Education/research generative-AI governance	No	Professional/normative guidance	General generative AI	Institutional governance	Student work/data	8. Institutional authorization, monitoring & incident response	Education institutions need human-centered, privacy-protective and pedagogically validated policies for GenAI rather than relying on public availability as evidence of educational suitability.	Public GenAI tools may be adopted faster than institutional validation, privacy protections and educator capacity can develop.	Privacy breaches, poorly aligned educational use, inequitable access, and loss of human agency in teaching and learning.	Multiple stakeholders	UNESCO's global guidance calls for data-privacy protection, human agency, age-appropriate and ethical validation, pedagogical design, and coherent policy frameworks for GenAI in education and research.	Require institutional validation proportional to risk; protect sensitive data; preserve human agency; align approved uses with educational purpose; maintain clear institutional guidance that can be updated as tools change.	Institution/medical school	No	Partial/indirect	Partial/indirect	High	4	5	5	4.6	UNESCO guidance overview: human-centered approach, data privacy, institutional preparedness/validation, coherent policy frameworks.	Institutional governance	S6 addition; authoritative education-level governance source.
69	Jin, 2025 Global Policies	Jin Y, Yan L, Echeverria V, Gašević D, Martinez-Maldonado R. Generative AI in higher education: A global perspective of institutional adoption policies and guidelines. Computers and Education: Artificial Intelligence. 2025;8:100348. doi:10.1016/j.caeai.2024.100348.	2025	International	https://doi.org/10.1016/j.caeai.2024.100348	Original empirical research	Published	Yes	Global higher-education GenAI adoption policy	No	Transferable HPE evidence	General generative AI	Institutional governance	No sensitive input	7. Human oversight & accountability	Successful institutional GenAI adoption depends on clear communication channels, stakeholder collaboration, explicit roles and responsibilities, attention to privacy/equity, and ongoing evaluation.	Policies may endorse AI in principle yet fail to define who decides, communicates, evaluates and revises practice.	Fragmented implementation, inconsistent local rules, inequitable adoption and unclear decision ownership.	Multiple stakeholders	Analysis of GenAI policies across 40 universities in six global regions found proactive but cautious adoption; gaps persisted in comprehensive policy frameworks, privacy and equitable access. The study emphasizes clear faculty/student/administrator responsibilities, communication and ongoing evaluation.	Assign role-specific responsibilities across learner, educator, course/assessment leadership and institution; define communication/escalation channels; review adoption outcomes periodically and revise policy as evidence/tools change.	Multiple actors	No	Partial/indirect	No	Moderate	4	5	5	4.6	Computers & Education: AI 2025;8:100348: 40 universities across six regions; clear roles, collaboration, privacy/equity and ongoing evaluation.	Accountability/oversight	S6 addition; supports multi-level role assignment instead of a single 'faculty responsible' rule.
70	Ganguly, 2025 Research Guidance	Ganguly A, Johri A, Ali A, McDonald N. Generative artificial intelligence for academic research: evidence from guidance issued for researchers by higher education institutions in the United States. AI and Ethics. 2025;5:3917-3933. doi:10.1007/s43681-025-00688-7.	2025	United States	https://doi.org/10.1007/s43681-025-00688-7	Original empirical research	Published	Yes	Higher-education GenAI research guidance	No	Transferable HPE evidence	General generative AI	Institutional governance	Public text	7. Human oversight & accountability	Institutional guidance commonly requires users to understand model limitations, provenance, privacy and IP, disclose GenAI use, follow external rules, and remain personally accountable for compliance.	Governance can become ineffective if responsibility is assigned to users without adequate institutional tools, training, approved resources or escalation mechanisms.	Unclear accountability, inconsistent disclosure, privacy/IP violations, and compliance failures.	Educator/faculty	Thematic analysis of guidance from 30 US R1 institutions found recurring expectations around model limitations, data provenance, authorship/attribution, privacy, IP, acknowledgement/disclosure, institutional training and individual researcher accountability.	Define the human owner for each AI-supported educational output; require disclosure/documentation appropriate to use; provide institutional training, approved-tool guidance and escalation routes rather than relying on personal responsibility alone.	Individual educator	No	No	Partial/indirect	Moderate	4	5	5	4.6	AI and Ethics abstract: 30 R1 institutions; provenance/model limitations/privacy/IP/disclosure; onus of compliance commonly placed on individual researchers.	Accountability/oversight	S6 addition; useful for human responsibility but also highlights the danger of overloading individuals with institutional risk.
71	McDonald, 2025 Policies	McDonald N, Johri A, Ali A, Collier AH. Generative artificial intelligence in higher education: Evidence from an analysis of institutional policies and guidelines. Computers in Human Behavior: Artificial Humans. 2025;3:100121. doi:10.1016/j.chbah.2025.100121.	2025	United States	https://doi.org/10.1016/j.chbah.2025.100121	Original empirical research	Published	Yes	Higher-education institutional GenAI policy	No	Transferable HPE evidence	General generative AI	Institutional governance	No sensitive input	8. Institutional authorization, monitoring & incident response	Institutional GenAI guidance is widespread but uneven and often generic, creating a gap between broad policy and discipline-specific operational controls.	Faculty may be given extensive general guidance without clear discipline-specific decision rules, increasing burden and inconsistent interpretation.	Inconsistent practices across courses, unclear expectations, and policy implementation that depends excessively on individual faculty judgment.	Educator/faculty	Analysis of 116 US R1 universities found 63% encouraged GenAI use, 41% offered detailed classroom guidance, 56% provided sample syllabi, and 50% provided curriculum/activities. The authors caution that guidance can become burdensome for faculty.	Translate institutional principles into concise discipline-specific use cases, risk tiers, responsible roles and escalation routes rather than adding another broad policy document.	Anatomy department	No	Partial/indirect	No	Moderate	4	5	5	4.6	NSF repository/journal record: 116 R1 institutions; 63% encourage use; 41% detailed guidance; 56% sample syllabi; 50% curriculum/activities.	Institutional governance	S6 addition; directly supports the need for an anatomy-level operational layer beneath university-wide policy.
72	ICMJE, 2026 AI	International Committee of Medical Journal Editors. Recommendations for the Conduct, Reporting, Editing, and Publication of Scholarly Work in Medical Journals: Use of Artificial Intelligence in Publishing. Updated January 2026.	2026	International	https://www.icmje.org/recommendations/browse/artificial-intelligence/	Professional guideline	Guidance/policy	No	Medical publishing/AI transparency and accountability	No	Professional/normative guidance	General generative AI	Resource generation	Public text	6. Provenance, IP & disclosure	AI use does not transfer responsibility from humans; users must ensure accuracy, attribution, confidentiality and transparent disclosure, and institutions/journals should have explicit AI policies.	AI can generate authoritative-sounding but incorrect, incomplete or biased content and can compromise confidentiality when sensitive material is uploaded to systems without assured protections.	Misinformation, plagiarism/attribution failures, confidentiality breaches and unclear responsibility.	Publisher/journal	The January 2026 ICMJE recommendations state that humans remain responsible for AI-assisted material, authors must disclose how AI was used, AI cannot be an author, users must verify accuracy/attribution, and confidential manuscripts should not be uploaded where confidentiality cannot be assured.	Carry the same accountability logic into anatomy education: the human approving AI-assisted material remains responsible; document material AI use where relevant; do not upload confidential/sensitive content to systems without appropriate protections and authorization.	Individual educator	Partial/indirect	No	Yes	High	4	5	5	4.6	ICMJE 2026 AI sections: disclosure, human responsibility, accuracy/attribution, confidentiality, journal policy.	Accountability/oversight	S6 addition; publishing guidance is not educational policy, so use as a strong accountability/disclosure precedent rather than a direct anatomy rule.
73	Alfredo, 2024 Human Centred	Alfredo R, Echeverria V, Jin Y, Yan L, Swiecki Z, Gašević D, Martinez-Maldonado R. Human-centred learning analytics and AI in education: A systematic literature review. Computers and Education: Artificial Intelligence. 2024;6:100215. doi:10.1016/j.caeai.2024.100215.	2024	International	https://doi.org/10.1016/j.caeai.2024.100215	Systematic review	Published	Yes	AI in education/human-centred system design	No	Transferable HPE evidence	Conventional AI (foundational only)	Institutional governance	Student work/data	8. Institutional authorization, monitoring & incident response	Human-centred educational AI requires stakeholder involvement, meaningful human control, safety/reliability/trustworthiness, and attention after launch; monitoring is commonly underdeveloped.	Educational AI projects often concentrate on design/evaluation before deployment and neglect the post-launch phase in which real-world harms and workflow failures emerge.	Poor fit with learner/educator needs, loss of user control, unrecognized failures after deployment, and weak trust.	Multiple stakeholders	A systematic review of 108 human-centred learning-analytics/AI-in-education studies found limited end-user involvement and reported that only 9% considered the launch-and-monitor phase. The authors recommend stakeholder involvement throughout design/deployment and balancing human control with automation.	Include learners/educators in evaluation where feasible; preserve meaningful human control; define post-deployment monitoring, feedback and review rather than treating initial validation as sufficient.	Institution/medical school	No	Partial/indirect	Partial/indirect	Moderate	4	5	5	4.6	Computers & Education: AI 2024;6:100215: 108 studies; only 9% addressed launch/monitor; recommends stakeholder involvement and human control.	Institutional governance	S6 addition; strong educational evidence for monitoring and stakeholder participation, though not GenAI/anatomy specific.
